# Hydraulic–physiological coordination predicts drought recovery: evidence from ten dry–hot valley species

**DOI:** 10.3389/fpls.2025.1715687

**Published:** 2025-12-15

**Authors:** Yunchen Zhang, Jianying Yang, Xu Yuan, Yandong Yang, Xiaodong Ji, Jinnan Ji, Yan Zhang, Jiao Huang

**Affiliations:** 1 College of Soil and Water Conservation, Beijing Forestry University, Beijing, China; 2 Huaneng Lancang River Hydropower Co., Ltd., Kunming, China; 3 Huaneng Lancang River Upstream Hydropower Co., Ltd., Changdu, China

**Keywords:** hot–dry valley, drought resistance, hydraulic traits, physiological recovery, principal component analysis, ecological restoration

## Abstract

**Introduction:**

Escalating drought in the Lancang River dry–hot valley demands trait-based rules for selecting planting material that can both persist through prolonged water deficits and rebound after rainfall.

**Methods:**

We conducted a controlled drought–rewatering experiment on ten native species (seven shrubs; three herbs) across a graded soil-water regime and quantified twenty-five functional traits spanning morphology, photosynthesis and photochemistry, biochemistry, hydraulics, and nutrient use.

**Results:**

Shrubs generally adopted a conservative strategy, exhibiting more negative xylem pressure at 50% loss of conductivity (P50), wider hydraulic safety margins, and faster recovery of PSII efficiency (Fv/Fm) after rewatering; herbs were more acquisitive, with higher specific leaf area and instantaneous water-use efficiency but reduced hydraulic safety. Trait-network analyses revealed hydraulic variables (P50, specific hydraulic conductivity, and turgor loss point) as central nodes tightly covarying with photosynthetic capacity and antioxidative activity, linking plant water status, carbon gain, and stress metabolism. Under severe drought, rising percent loss of conductivity and increased non-photochemical quenching delineated failure domains in which hydraulic disconnection and photoprotective energy dissipation jointly constrained function. Rewatering improved leaf water status and photochemistry but recovery trajectories were species-specific and retained legacy effects consistent with safety–efficiency trade-offs. Multivariate ordination and integrated scoring separated species into tolerant, intermediate, and sensitive types, with the composite ranking highlighting *Rumex hastatus*, *Caryopteris forrestii*, and *Sophora davidii* as priority candidates that couple high hydraulic safety with resilient photosynthetic recovery.

**Discussion/Conclusion:**

These findings show that drought performance in this extreme environment emerges from hydraulic–physiological coordination balancing safety, efficiency, and resilience. Practically, they support a minimal diagnostic panel for rapid screening—P50, turgor loss point, hydraulic safety margin, and post-rewatering Fv/Fm recovery—supplemented by acquisitive leaf traits to resolve strategy space, providing transferable criteria for restoration in drylands facing intensifying hydroclimatic variability.

## Graphical Abstract

**Figure:**
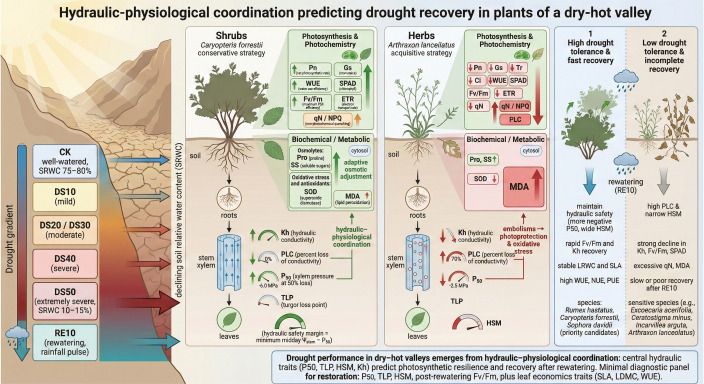

## 1 Introduction

Rising global temperatures are amplifying the frequency, duration, and intensity of drought, with the most acute impacts concentrated in hot–dry regions where plants experience chronic water limitation (Solomon et al., 2007). Dry–hot valleys provide a tractable natural model for testing drought‐adaptation hypotheses because they combine persistently high temperature, low precipitation, strong foehn effects, steep topography, and long-term soil aridity (Li et al., 2025; Sun et al., 2025). Within this class of systems, the Lancang River dry–hot valley couples high biodiversity with pronounced ecological degradation, including the contraction of native evergreen oak forests and severe soil erosion (erosion modulus 5,200–8,600 t·km^-2^·yr^-1^) (Zhang et al., 2023; Li et al., 2023a). These vegetation and soil losses interact with climatic stress to reinforce a “drought pressure–vegetation decline–soil function loss” cycle, underscoring the need for trait-based species selection to stabilize ecosystem processes in this landscape and in hot–dry ecosystems more broadly.

Over the past decade, drought‐resistance research has moved from single indicators toward multi-trait, multi-scale frameworks that emphasize coordinated adjustments among structural and functional traits (Poorter et al., 2012; Cornelissen et al., 2003). A central axis emerging from this work is hydraulic safety, commonly indexed by the water potential at 50% loss of xylem conductivity (P50) (Choat et al., 2012) and grounded in process-based hydraulics (Sperry and Love, 2015). Complementary physiological indicators track stress to the photosynthetic apparatus: the maximum quantum efficiency of PSII (Fv/Fm) and antioxidant enzyme activity such as superoxide dismutase (SOD) capture photochemical integrity and metabolic buffering, respectively (Hu et al., 2009; Takahashi and Badger, 2009). Life-form contrasts further suggest divergent routes to drought tolerance: shrubs tend to elevate hydraulic safety via adjustments that reduce embolism risk, whereas herbs often exploit episodic rainfall pulses with acquisitive leaf economics (Maherali et al., 2004; Wright et al., 2004). These themes have been organized along trait axes related to allocation (e.g., height, root:shoot ratio, LDMC, SLA), carbon–water exchange (e.g., Pn, Gs, WUE, Fv/Fm, qN), biochemical buffering (e.g., SOD, MDA), and hydraulic operation (e.g., Kh, PLC, TLP, HSM), providing an integrative lens on resistance–recovery strategies (Skelton et al., 2015, 2021). Recent syntheses have expanded leaf–hydraulic linkages and cross-scale trait coordination under aridity (Scoffoni and Sack, 2023; Moreno et al., 2024), while drought-related mortality risk has been framed as a coupled physiological syndrome (McDowell et al., 2011). At the ecosystem scale, changing stress–facilitation balances along aridity gradients further shape community assembly and performance (Michalet et al., 2014). Despite this progress, important gaps remain for hot–dry valleys: empirical tests that explicitly couple hydraulic damage and photochemical regulation across full drought gradients are scarce (Li et al., 2017); structural mediation of hydraulic safety via allocation traits such as root:shoot ratio remains under-quantified (Duursma et al., 2014); the joint evaluation of resistance during stress and resilience after rewatering is rarely done on the same individuals (Whitmore and Whalley, 2009); and life-form contrasts under extreme drought require a unified, mechanistic comparison to inform restoration (Cai et al., 2023; Li et al., 2017).

We therefore adopt a pot-based, full-gradient drought design followed by rewatering to generate mechanistic, comparable evidence across species and life forms. Controlled water regulation allows precise manipulation of drought intensity and duration, isolating climatic forcing from confounding topography and foehn variability that characterize dry–hot valleys (Li et al., 2025; Sun et al., 2025). This control resolves nonlinear responses and thresholds in hydraulic vulnerability (e.g., P50 and PLC), which are otherwise difficult to separate from site heterogeneity (Choat et al., 2012; Sperry and Love, 2015). The design also enables repeated, paired measurements of photochemistry and metabolism (e.g., Fv/Fm, qN, SOD) on the same individuals, directly linking physiological regulation to hydraulic status through time (Hu et al., 2009; Takahashi and Badger, 2009). Standardized conditions facilitate acquisition of allocation and leaf economics traits (e.g., root:shoot ratio, LDMC, SLA) for cross-species comparisons relevant to drought strategy (Poorter et al., 2012; Cornelissen et al., 2003; Maherali et al., 2004; Wright et al., 2004). Critically, the rewatering phase quantifies resilience—i.e., recovery of Fv/Fm and hydraulic function (Kh)—which is essential to understand post-stress performance and mortality risk under fluctuating hydroclimates (Whitmore and Whalley, 2009; McDowell et al., 2011; Skelton et al., 2015, 2021; Scoffoni and Sack, 2023; Moreno et al., 2024). Measurements of TLP and related water relations further anchor comparisons of hydraulic safety margins across life forms (Bartlett et al., 2012; Skelton et al., 2015).

Guided by the gaps above, we ask three questions. (i) Do shrubs and herbs in a hot–dry valley follow distinct but coordinated hydraulic–physiological strategies under extreme drought? (ii) Are cross-scale relationships consistent with a “hydraulic safety margin–physiological resilience” hypothesis—specifically, does structural allocation mediate hydraulic safety and do hydraulic losses predict photochemical down-regulation and recovery after rewatering? (iii) Can a multidimensional, trait-based classification that integrates morphology, physiology, and hydraulics provide actionable guidance for species deployment in dry–hot valley restoration?

In a controlled pot experiment, we quantified twenty-five traits spanning morphology, photosynthesis/photochemistry, biochemistry, and hydraulics for ten native species from the Lancang River dry–hot valley (seven shrubs, three herbs) across a full water-regulation gradient (control, mild drought, severe drought) followed by rewatering (Zhang et al., 2023; Li et al., 2023a). To synthesize multivariate trait covariation and link species responses to drought intensity, we used principal component analysis (PCA) and fuzzy-membership functions to derive composite drought-tolerance scores, and redundancy analysis (RDA) to assess trait–environment relationships (Ziegler et al., 2019; Yan et al., 2020). In summary, this study not only provides theoretical support and species selection criteria for the ecological restoration of the Lancang River dry-hot valley, but also offers a reference for the adaptive management of arid ecosystems worldwide.

## 2 Materials and methods

### 2.1 Study area

The study was conducted in the hot–dry valleys of the Lancang River spanning Deqin County (Diqing Tibetan Autonomous Prefecture) and Mangkang County, Yunnan Province, China (98°049′–98°830′ E, 28°541′–29°718′ N). Situated in the core of the Hengduan Mountains—an important north–south geomorphological corridor on the southeastern Tibetan Plateau—this region forms a key ecological observation area within the upper Lancang–Mekong transboundary basin. A linear survey transect extended from Gushui Town northward along National Highway G214 through Foshan, Muxu, and Quzika Townships to Rumei Town. The landscape comprises steep alpine gorges with an exceptional altitudinal range (~2,000–6,740 m at Mount Kawagebo), giving rise to the local “ten microclimates within ten miles” phenomenon; mean slopes exceed 35%. The climate is a typical plateau monsoon regime, with an annual mean temperature of 4.7°C, precipitation of 600–800 mm, and total annual sunshine of 1,980.7 h. Orographic effects combined with Tibetan Plateau uplift and the southwest monsoon generate pronounced hydroclimatic seasonality: >70% of precipitation falls during a short rainy season (June–September), while the prolonged dry season (November–April) is dominated by strong foehn winds; during this period, potential evapotranspiration exceeds precipitation by more than threefold, leading to persistent soil moisture deficits. Vegetation exhibits clear altitudinal zonation: hot–dry valley shrublands at 2,000–3,000 m (dominated by *Vitex negundo*, *Sophora davidii*, *Elsholtzia capituligera*), transitional mixed needle–broadleaf forests at 3,000–4,000 m (including *Quercus aquifolioides*, *Picea likiangensis*), and subalpine coniferous forests (e.g., *Abies georgei*) with alpine meadows above 4,000 m. Scattered populations of *Taxus wallichiana* and *Cupressus gigantea*—both nationally protected in China—underscore the region’s high conservation value.

### 2.2 Experimental materials

In December 2023, seeds of ten native species typical of the Lancang River dry–hot valleys—*Caryopteris forrestii*, *Elsholtzia capituligera*, *Vitex negundo*, *Sophora davidii*, *Rumex hastatus*, *Ceratostigma minus*, *Excoecaria acerifolia*, *Incarvillea arguta*, *Artemisia vestita*, and *Arthraxon lanceolatus*—were collected and prepared for cultivation. All seed lots satisfied preset quality thresholds upon receipt (purity ≥ 98%; moisture ≤ 10%). After sun-drying for three consecutive days (10:00–14:00), seeds were surface-sterilized in 0.1% HgCl_2_, pre-germinated by soaking at 25 °C, and sown into standardized 6.0 L polyethylene pots filled with the common growth substrate used in this study (16 pots per species; total n = 160). Pretreatments were adjusted to seed type: (i) herbaceous species (e.g., *Incarvillea arguta*, *Artemisia vestita*) were soaked in warm water (40 ± 2 °C) for 4 h and sown at 1–3 × seed-diameter depth; (ii) non-hard-coated legumes/shrubs (e.g., *Sophora davidii*) were soaked in warm water for 12 h until swollen, incubated on moist tissue at 25 °C to radicle emergence, and sown at 2–3 × seed-diameter depth; and (iii) hard-coated seeds (e.g., *Excoecaria acerifolia*) were treated with a 3,000-fold GA_3_ solution (≈ 33.3 mg L^-1^) for 24 h to break dormancy, germinated on moist tissue to radicle protrusion, and sown at ≈ 3 × seed-diameter depth. Seedlings were maintained in a controlled greenhouse (photoperiod 14 h light/10 h dark; 25/18 °C day/night). At 30 days after emergence, stands were thinned to eight uniform seedlings per pot for subsequent experiments. A summary of species and material characteristics is provided in **Table 1**.

**Table 1:** Overview of experimental plant varieties.

Plant name	Abbreviation	Family	Genus	Plant type
*Caryopteris forrestii* Diels	Cf	*Verbenaceae*	*Elsholtzia*	Shrub
*Elsholtzia capituligera* C. Y. Wu	Ec	*Verbenaceae*	*Elsholtzia*	Subshrub
*VitexnegundoL.* var.*microphyllaHand.-*Mazz.	Vn	*Verbenaceae*	*Vitex*	Shrub
*Sophora davidii* Kom. ex Pavol.	Sd	*Fabaceae*	*Sophora*	Shrub
*Rumex hastatus* D. Don	Rh	*Polygonaceae*	*Rumex*	Herb
*Ceratostigma minus* Stapf ex Prain	Cm	*Plumbaginaceae*	*Ceratostigma*	Small shrub
*Excoecaria acerifolia* Didr.	Ea	*Euphorbiaceae*	*Excoecaria*	Shrub
*Incarvillea arguta* (Royle) Royle	Ia	*Bignoniaceae*	*Incarvillea*	Herb
*Artemisia vestita* Wall. ex Bess.	Av	*Asteraceae*	*Artemisia*	Herb
*Arthraxon lanceolatus* (Roxb.) Hochst.	Ai	*Poaceae*	*Arthraxon*	Shade herb

The plant names in the result analysis are all represented by abbreviations.

Initial seedling specifications at the onset of drought treatments. To report size and woodiness at the start of water-regulation treatments (Day 0), we recorded seedling height and stem diameter. Because all individuals were < 1.3 m tall, diameter at breast height (DBH) is not applicable; instead, we report basal stem diameter (measured 1 cm above the substrate) as the standard proxy for seedlings. Across the six shrub species, seedlings averaged 7–28 cm in height (species means) with basal stem diameter 1.2–3.8 mm. Across the four herb species, seedlings were 5–20 cm tall; stem diameter was < 1 mm and therefore not quantified. Developmental status at treatment onset was as follows: shrub stems were woody (lignification with incipient secondary thickening evident), whereas herbaceous stems remained non-woody.

### 2.3 Experimental design

To unify the initial moisture state, a pre-treatment was conducted at the end of April 2025: all pots received continuous saturated irrigation for 3 days until soil relative water content (SRWC) ≥ 95%. Day 0 was defined as the morning immediately after pre-saturation.

A complete water-treatment system was then established and maintained for 50 consecutive days (Day 0–Day 50): (i) a well-watered control (CK), irrigated every 3 days to keep SRWC at 75–80%; and (ii) a five-level drought gradient defined by SRWC targets and held constant throughout the stress period—mild drought, SRWC 60–65%; moderate drought, SRWC 40–45% and 30–35%; severe drought, SRWC 20–25%; and extremely severe drought, SRWC 10–15%. A single rewatering treatment (RE) was imposed once, immediately after the 50-day drought phase (on Day 50), by restoring SRWC to the CK target (75–80%) to simulate rainfall recharge. Plants were then monitored for an additional 10 days (Day 50–Day 60), after which the experiment was terminated (final endpoint at Day 60, denoted RE10).

Moisture was managed and verified daily at 18:00 using a time-domain reflectometry (TDR) probe; SRWC targets were maintained by quantitative watering based on the measured values. For each species × treatment combination, five biological replicates (pots) were used (n = 5) to ensure statistical power and repeatability.

Functional, disease-free leaves from the upper–middle canopy were collected every 10 days during the drought phase on fixed timepoints—Day 10, 20, 30, 40, and 50—and once at the end of the 10-day recovery (Day 60). Immediately after collection, samples were snap-frozen in liquid nitrogen and stored at −80°C for subsequent physiological and biochemical assays. To avoid ambiguity, “DSxx” denotes the day of stress sampling timepoints (DS10, DS20, DS30, DS40, DS50 = Days 10, 20, 30, 40, 50), whereas drought severity levels are defined exclusively by the SRWC target ranges above. “RE10” denotes 10 days after the single rewatering event (Day 60). CK plants were measured on the same calendar days as the drought treatments.

### 2.4 Measurements and indicators

#### 2.4.1 Soil water content

Field capacity (FC) was used as the reference for drought-stress classification (Luo et al., 2021). FC was determined with the core (ring-knife) method at a soil depth of 0–15 cm. Following full saturation by irrigation, pots were sealed with an evaporation-barrier film to suppress surface water loss and allowed to drain under gravity for 48 h. Intact soil cores (100 cm³) were then collected and oven-dried at 105°C to constant mass. FC was calculated according to Equation 1 (Yuan et al., 2023):

(1) $$\begin{array}{c} \text{FC}\;(\%)=(\text{Weight of saturated soil}\\ -\text{Dry soil weight})/\text{Dry soil weight}\times 100\% \end{array}$$

Soil water dynamics were monitored using a three-layer array of EC-5 probes installed at 0–5, 5–10, and 10–15 cm. For each instrumented pot, three probes per layer were positioned 120° apart around the plant base (i.e., nine probes per pot). After installation, the surrounding soil was gently compacted and allowed to equilibrate for 72 h to ensure adequate sensor–soil contact. We instrumented one drought-treated pot per species (10 species × 1 pot × 9 probes = 90 probes in total). Control (CK) pots were kept at high moisture and were not instrumented with EC-5 arrays. For each depth layer within an instrumented pot, the arithmetic mean of the three parallel probes (after outlier exclusion) was used as the layer value.

**Figure 1:**
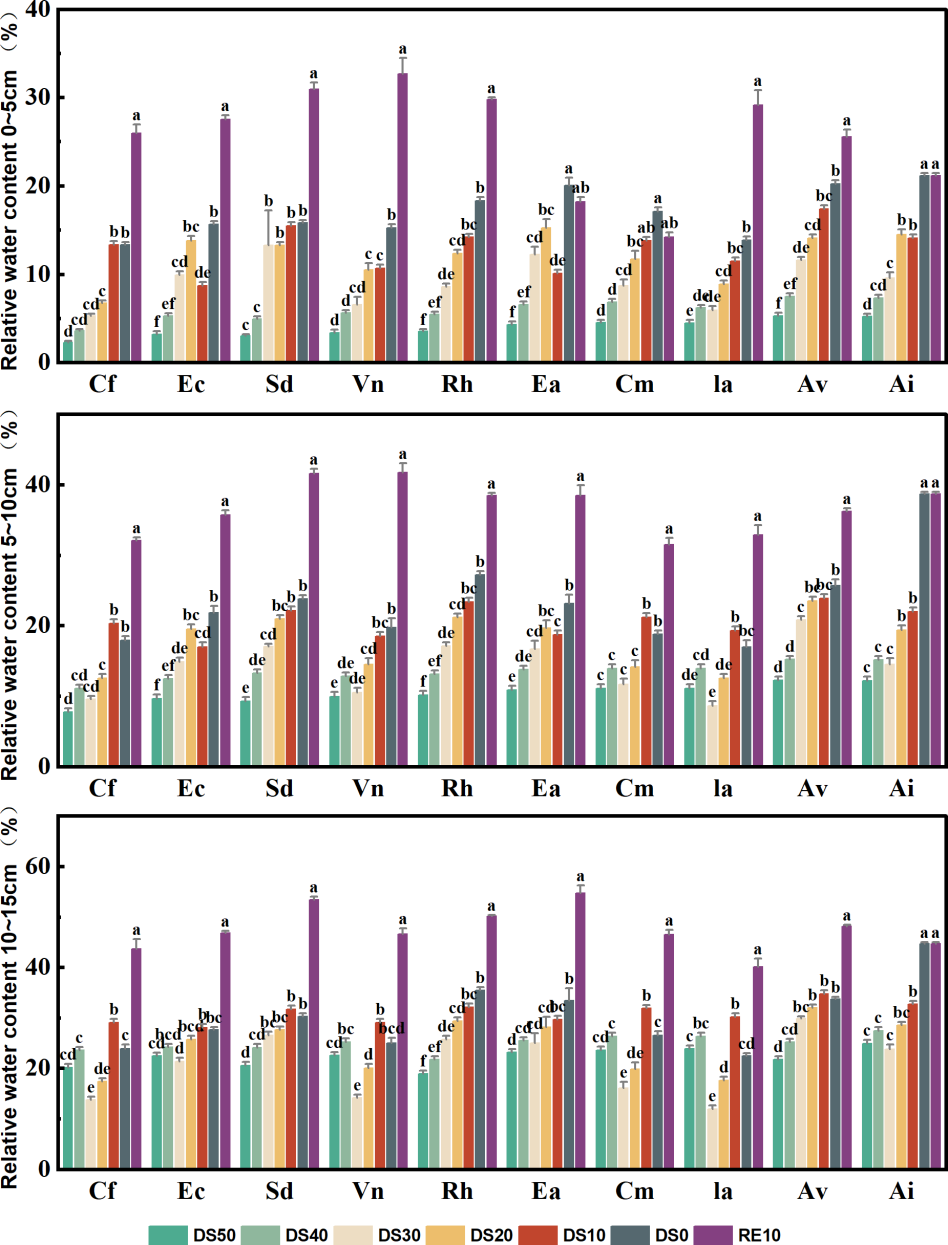
Spatial and temporal dynamics of soil moisture during drought stress rehydration process. Mild drought (DS10): SRWC range of 60% -65%; Moderate drought (DS20 and DS30): SRWC range of 40% -45% and 30% -35%; Severe drought (DS40): SRWC ranges from 20% to 25%; Extremely severe drought (DS50): SRWC range of 10% -15%; Rehydration treatment (RE10): SRWC is between 75% and 80%. Cf represents *Caryopteris forrestii*; Ec represents *Elsholtzia capituligera*; Sd represents *Sophora davidii*; Vn represents *Vitex negundo*; Rh represents Rumex hash status; Ea represents *Excoecaria acerifolia*; Cm represents *Ceratostigma minus*;Ia represents *Incarvilliea arguta*; Av represents *Artemisia vestita*; Ai represents *Arthraxon lanceolatus*. The following results analyze plant names using abbreviations instead.The drought gradient and Latin annotations of plants in **Figures 2**-**8** are consistent with **Figure 1**, the same applies below. The different letters in the figure indicate statistically significant differences (P<0.05) between groups under each drought stress gradient treatment, while the same letters indicate no significant differences between groups.

Monitoring with EC-5 arrays commenced on 10 May 2025 and proceeded at 10-day intervals throughout the drought–rewatering cycle to generate the depth-resolved time series summarized in **Figure 1**. In parallel with these scheduled readings, daily SRWC management and verification were performed using TDR at 18:00 to maintain target ranges (see Section 2.3). Soil relative water content (SRWC) was calculated as Equation 2:

(2) SRWC(%)=Measured soil moisture/FC×100%

#### 2.4.2 Leaf functional traits

We quantified four structural traits—plant height (H), leaf relative water content (LRWC), leaf dry matter content (LDMC), and specific leaf area (SLA)—on days 10, 20, 30, 40, and 50 of the drought treatment and again 10 days after rewatering, for both control (CK) and drought-stress (DS) groups across all ten species. Plant height was measured vertically with a steel ruler (0.1 cm resolution). Above- and belowground biomass were harvested separately; fresh mass (FW) was recorded immediately after cutting and gentle cleaning using an analytical balance (0.01 g resolution). Samples were then heated at 105°C for 30 min to inactivate enzymes and oven-dried at 80°C for 24–48 h to constant mass, defined as a change < 0.01 g across two consecutive weighings (Poorter and Garnier, 1999) The Equation 3 is:

(3) Dry-fresh ratio(%)=DW/FW×100%

Functional leaves (healthy, fully expanded, mid-upper canopy) were selected. FW was recorded, leaf area (LA) was measured using a Li-3000A area meter, saturated fresh weight (SFW) was obtained after 24 h in deionized water, and DW was determined after oven-drying at 65°C.

Equations 4–6 is (Wright et al., 2001):

(4) LRWC(%)=(FW−DW)/(SFW−DW)×100%

(5) LDMC=DW/FW

(6) $$\text{SLA}({\mathrm{cm}}^{2}/\text{g})=\text{LA}/\text{DW}$$

All measurements followed standardized protocols to ensure consistency and comparability.

#### 2.4.3 Physiological and biochemical traits

At each drought and rewatering stage, functional leaves from all treatment groups were collected, immediately snap-frozen in liquid nitrogen, and stored at −80°C until analysis. For biochemical assays, 0.5 g of frozen leaf tissue per replicate was ground to a fine powder and homogenized in ice-cold 50 mmol L^-1^ phosphate buffer (pH 7.8). The homogenate was clarified by centrifugation at 12,000 rpm for 20 min at 4 °C, and the resulting supernatant was used for subsequent measurements. SOD activity: NBT photochemical reduction inhibition method; MDA content: Thiobarbituric acid (TBA) colorimetric assay; Proline content: Sulfosalicylic acid-based colorimetric method; Soluble sugar (SS) content: Anthrone–sulfuric acid method (Wilson et al., 1999).

Chlorophyll content was measured using a SPAD-502 meter. All treatments were performed with three biological replicates per time point.

#### 2.4.4 Photosynthetic parameters

Gas exchange was measured on days 10, 20, 30, 40, 50, and 60 of the drought treatment between 09:00 and 11:00. For each treatment and species, ten pots were selected, and three fully expanded, similarly sun-exposed leaves were sampled per plant (three replicates). Measurements were taken with a LI-6400XT portable photosynthesis system (LI-COR, USA), recording net photosynthetic rate (Pn), stomatal conductance (Gs), transpiration rate (Tr), and intercellular CO_2_ concentration (Ci) under standardized conditions: PPFD = 1000 μmol m^-2^ s^-1^, reference CO_2_ = 380 ± 5 μmol mol^-1^, and leaf temperature = 25 ± 1 °C. Data interpretation followed the Equation 7 (Laughlin et al., 2009):

(7) Water Use Efficiency(WUE)=Pn/Tr

#### 2.4.5 Chlorophyll fluorescence parameters

Chlorophyll fluorescence was assessed with a LI-6400XT fluorometer chamber (LI-COR, USA). Mature, fully expanded leaves were dark-adapted for 30 min, after which initial fluorescence (Fo) was recorded under a weak modulated measuring beam (630 nm). A saturating pulse (6,000–10,000 μmol m^-2^ s^-1^, 0.8 s) was then applied to obtain maximum fluorescence in the dark-adapted state (Fm). Leaves were subsequently exposed to actinic light (photosynthetic photon flux density, PPFD = 1,000 μmol m^-2^ s^-1^) to reach a steady state, at which steady fluorescence (Fs) was recorded and a second saturating pulse applied to determine maximum fluorescence in the light (Fm′); a brief far-red illumination was used to estimate minimal fluorescence in the light (Fo′). Key parameters were calculated as follows: variable fluorescence, Fv = Fm−Fo; maximum photochemical efficiency of PSII, Fv/Fm; effective quantum yield of PSII, ΦPSII = (Fm′−Fs)/Fm′; photochemical quenching, qP = (Fm′− Fs)/(Fm′−Fo′); non-photochemical quenching, NPQ = (Fm−Fm′)/Fm′; and electron transport rate, ETR = ΦPSII × PPFD × 0.84 × 0.5. Environmental settings were controlled (25 ± 1 °C, CO_2_ = 380 ± 5 μmol·mol^-1^). All measurements were performed in triplicate (Anderegg et al., 2018).

#### 2.4.6 Nutrient use efficiency

Independence from the drought trial. The fertilization experiment was conducted on a separate cohort of plants under well-watered conditions and did not overlap with, nor interfere with, the drought–rewatering experiment described in Section 2.3. Throughout this nutrient trial, soil relative water content (SRWC) was maintained at 75–80% (CK target range); no drought gradients or rewatering events were applied.

Ten species were cultivated in a uniform, nutrient-free substrate. For each species, thirty uniform, healthy individuals were randomly assigned to two treatments—control (no fertilizer) and fertilization—with three replicates per treatment and five plants per replicate. The fertilized treatment received 100 mg N kg^-1^ and 60 mg P kg^-1^ (soil dry-mass basis); the control received no fertilizer. Sampling was conducted on Days 0, 10, 20, 30, 40, 50, and 60. At each time point, two plants per replicate were harvested and separated into roots, stems, and leaves, oven-dried at 65 °C, and weighed. Dried tissues were finely ground and passed through a sieve for chemical analyses: Total N by the Kjeldahl method and Total P by the vanadomolybdate yellow colorimetric method (Osnas et al., 2018).

Efficiency indicators are shown in Equations 8, 9:

(8) $$\begin{array}{c} \text{Nitrogen Use Efficiency}(\text{NUE},\;\%)\\ =(\text{Total N uptake}/\text{N applied})\times 100\% \end{array}$$

(9) $$\begin{array}{c} \text{Phosphorus Use Efficiency}(\text{PUE},\;\%)\\ =(\text{Total P uptake}/\text{P applied})\times 100\% \end{array}$$

#### 2.4.7 Hydraulic traits

Sampling and segment preparation. For each species, at least five healthy individuals were sampled. Stems were cut predawn and immediately recut under water to avoid air entry. Straight stem segments were prepared with final segment length ≥ 1.5× the species’ maximum vessel length (MVL) to minimize open-vessel artefacts (Meziane and Shipley, 1999). MVL was determined in preliminary tests (dye perfusion on excised stems/roots), and segment length was then set accordingly; as a result, shrub segments were typically 30–40 cm and herbaceous segments 15–20 cm. When herb stems were hollow or excessively pithy, basal stem or primary-root segments containing continuous vascular bundles (primary xylem) were used to characterize conductive-tissue vulnerability in a manner comparable to woody stems.

Hydraulic conductivity (Kh), maximum conductivity (Kmax), and PLC. Native hydraulic conductivity (Kh) was measured with a XYL’EM-Plus low-pressure flow meter (Lab Scientific, France) under a driving pressure of 5–10 kPa using 1 mmol L^-1^ CaCl_2_ solution (pH 6.0, 0.22 μm filtered, degassed). Background flow was checked and subtracted where necessary; temperature was held at 25 ± 1 °C. The same segment was then flushed at 200 kPa with the measurement solution until flow stabilized to obtain Kmax. Percent loss of conductivity (PLC) was calculated as Equation 10:

(10) PLC(%)=[1−Kh/Kmax]×100%

To avoid the positive bias sometimes associated with certain pressure methods, vulnerability curves were generated with the centrifuge approach. Segments were subjected to stepwise xylem tensions from ≈ 0.5 to 4.5 MPa in 0.2–0.3 MPa increments. After each step, segments were removed, Kh was re-measured on the XYL’EM-Plus, and PLC–water potential (Ψ) pairs were obtained. A two-parameter Weibull function was fitted to the PLC–Ψ relationship to estimate the xylem pressure at 50% conductivity loss (P50). We report P50 as negative MPa values (more negative = greater embolism resistance). All species were processed with the same protocol, ensuring comparability across shrubs and herbs.

TLP was determined once per species by pressure–volume (P–V) analysis. Fully hydrated, mature leaves were bench-dehydrated in ~0.1 MPa Ψ increments; Ψ was measured with a pressure chamber, relative water content (RWC) was calculated gravimetrically, and TLP was identified as the breakpoint (inflection) on the Ψ–RWC curve derived from the linearized segments.

Because the pressure chamber directly measures leaf water potential, stem water potential (Ψstem) was estimated with the bagged-leaf equilibration method: target leaves were wrapped in foil (light-tight) for 2 h before measurement to equilibrate with the subtending stem xylem. Midday stem water potential was measured near solar noon (12:00–13:00). For the purposes of this experiment, the hydraulic safety margin was computed as Equation 11:

(11) $${\text{HSM}}_{\text{exp }}={\text{Ψ}}_{\text{stem}-\text{midday}(\text{min})\;}-{\text{P}}_{50}$$

here Ψstem–midday(min) is the lowest midday Ψstem recorded during the trial window, which occurred on Day 50 (the driest day under DS50). Thus, HSMexp represents the safety boundary for this experimental period; under longer observation windows, the minimum water potential—and therefore HSM—may differ.Trait treatment over time. Consistent with their biological interpretation, P50 and TLP were treated as species-level, time-invariant traits over the 60-day study (short-term drought is not expected to shift these thresholds). Any small repeated-measure fluctuations were considered within measurement error and were not interpreted as real temporal change. Time-varying drought exposure was therefore captured by Ψstem–midday, and HSMexp reflects the interaction between static hydraulic thresholds (P50) and the observed minimum operating water status during the imposed drought.

To minimize artefacts, segments were recut under water immediately prior to mounting; connectors were leak-tested; solutions were filtered/degassed; open-vessel risk was controlled by enforcing length ≥ 1.5× MVL; and all steps (tension sequence, solution, temperature, stabilization criteria) were standardized across species.

### 2.5 Integrated drought resistance evaluation

Given the multidimensional physiological and ecological responses to drought, no single trait can reliably represent drought resistance. Accordingly, we evaluated resistance using a suite of 25 indicators spanning structural, physiological, photosynthetic, hydraulic, and nutrient dimensions. All variables were first standardized by Z-score transformation to remove scale effects, and data suitability for dimension reduction was verified with Kaiser–Meyer–Olkin (KMO) and Bartlett’s tests. Principal component analysis (PCA) was then applied to identify the major orthogonal axes of trait covariation and to summarize species responses. To improve classification robustness and comparability across traits, we further employed fuzzy membership functions from fuzzy mathematics to map raw trait values to unitless membership scores in the range 0–1 (Poorter and Remkes, 1990), which were subsequently integrated to derive a comprehensive drought-resistance assessment.

For positively correlated traits, calculate using Equation 12:

(12) $$\text{R}({\text{X}}_{i})=({\text{X}}_{i}-{\text{X}}_{imin})/({\text{X}}_{imax}-{\text{X}}_{imin})$$

For negatively correlated traits, calculate using Equation 13:

(13) $$\text{R}({\text{X}}_{i})=1-[({\text{X}}_{i}-{\text{X}}_{imin})/({\text{X}}_{imax}-{\text{X}}_{imin})]$$

where X*_i_* is the observed value for species *i*, and X*_imax_* and X*_imin_* are the maximum and minimum values across all species. The average of the 25 membership values represented the composite drought resistance score.

### 2.6 Data processing and statistical analysis

Data were assessed for normality (Shapiro–Wilk) and homogeneity of variances (Levene’s test). When assumptions were met, one-way ANOVA (α = 0.05, two-tailed) followed by least significant difference (LSD) *post hoc* comparisons was applied. Baseline data processing was conducted in Microsoft Excel 2021; inferential statistics were performed in IBM SPSS Statistics 26.0; and figures were produced in OriginPro 2021 (OriginLab, USA). Associations among non-hydraulic drought-related traits were evaluated using Spearman’s rank correlation. For hydraulic traits, pairwise correlation matrices (and corresponding visualizations) were computed in R. Redundancy analysis (RDA) was implemented in Canoco 5.0 to quantify the variance in multidimensional drought responses explained by hydraulic predictors.

## 3 Results and analysis

### 3.1 Spatial and temporal dynamics of soil moisture during drought stress rehydration process

As shown in **Figure 1**, To transparently quantify drought intensity and validate gradient separation, we continuously monitored soil relative water content (SRWC, %) in three layers (0–5 cm, 5–10 cm, 10–15 cm) using a time-domain reflectometry (TDR) probe. **Figure 1** summarizes SRWC trajectories for all ten species across the drought–rewatering cycle.

During the 50-day drought phase, treatment targets were maintained as designed: CK at 75–80% SRWC; mild drought at 60–65%; moderate drought at 40–45% and 30–35%; severe drought at 20–25%; and extreme drought (DS50) at 10–15%. At the end of the drought phase (DS50), DS50 pots remained at 10–15% across depths, whereas CK stayed at ~75–80%. After the single rewatering event, SRWC recovered toward the CK range, reaching ~70–80% by Day 60 (RE10).

SRWC consistently decreased with shallower depth. The 0–5 cm layer exhibited the lowest values and the steepest declines under increasing drought severity, indicating that surface soil is most vulnerable to evaporative and plant-driven depletion. Mid (5–10 cm) and deeper (10–15 cm) layers retained higher SRWC than the surface but showed clear downward trends along the drought gradient.

While all species conformed to the imposed SRWC targets, shallow-layer differences were most pronounced: species coded *Excoecaria acerifolia*, *Ceratostigma minus*, *Artemisia vestita*, and *Incarvillea arguta* tended to maintain comparatively higher SRWC in the 0–5 cm layer across drought levels, whereas *Caryopteris forrestii*, *Elsholtzia capituligera*, and *Sophora davidii* showed lower values. In the 5–10 cm and 10–15 cm layers, interspecific differences persisted but were less marked than at the surface, consistent with deeper water access mitigating depletion. Collectively, these sensor-based data confirm that (i) the drought gradient was achieved and stable over time, (ii) depth effects were systematic (surface ≪ mid ⪅ deep), and (iii) species-level patterns observed in plant responses can be interpreted against a quantified, layer-resolved moisture background.

### 3.2 Changes in plant functional traits during drought stress rehydration process

**Figure 2:**
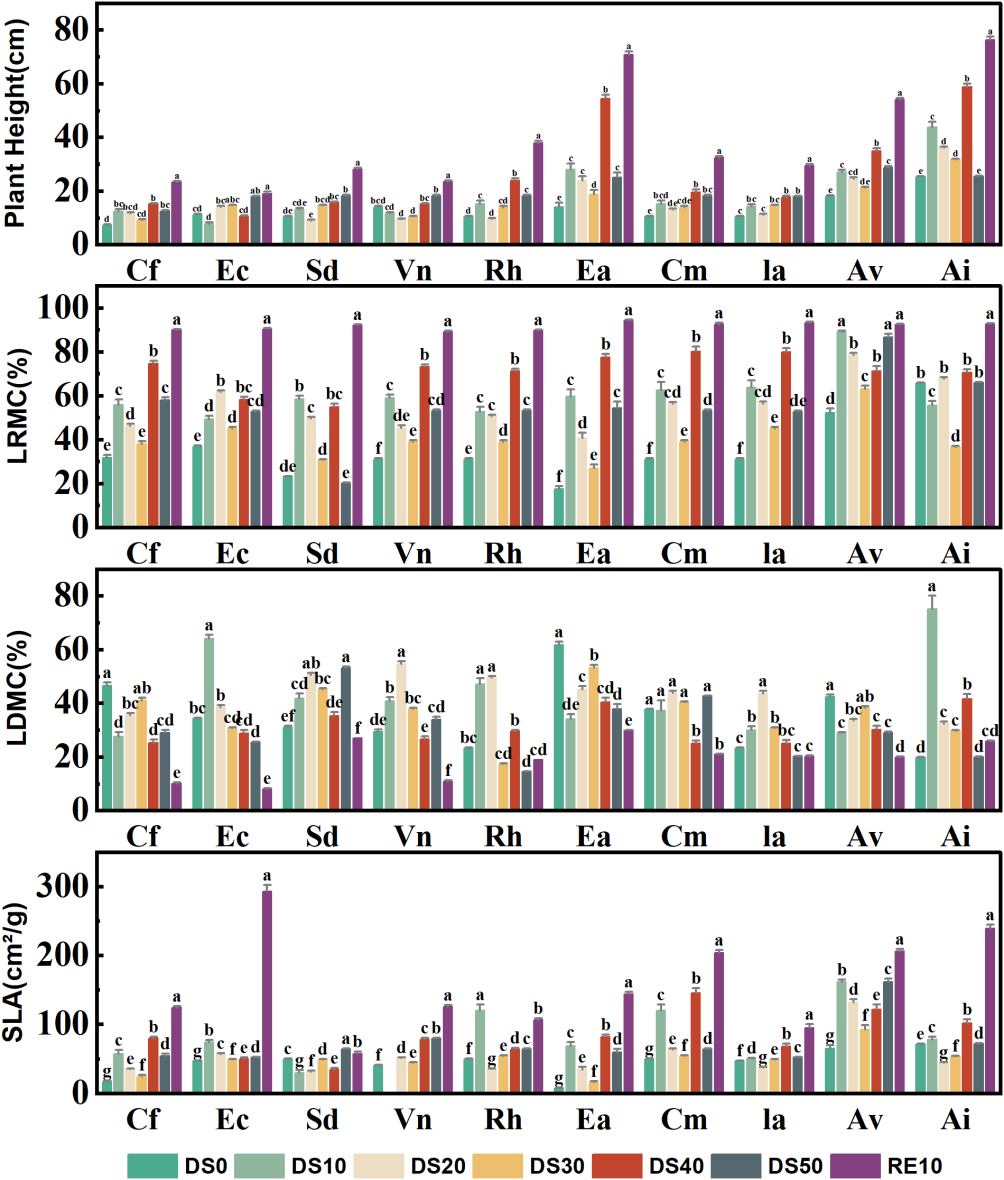
Changes in plant functional traits during drought stress rehydration process. The different letters in the figure indicate statistically significant differences (P<0.05) between groups under each drought stress gradient treatment, while the same letters indicate no significant differences between groups.

As shown in **Figure 2**, all measured plant functional traits—plant height (H), leaf relative water content (LRWC), leaf dry matter content (LDMC), and specific leaf area (SLA)—exhibited significant species-specific variation across drought–rewatering treatments (P < 0.05), reflecting divergent drought-resistance strategies. Drought suppressed vertical growth in most species, whereas rewatering generally facilitated partial recovery. At the end of the drought phase (DS50) under the extreme-drought treatment (SRWC 10–15%), *Excoecaria acerifolia* showed a marked reduction in height to ≈ 22 cm, which is ~39% of its post-rewatering value (RE10 ≈ 56 cm) and lower than its transient value at DS40 (≈ 36 cm). This decrease does not imply “shrinkage” of intact stems; rather, it reflects apex dieback and terminal twig necrosis under severe water deficit, which lowered the highest living point used to define plant height (from the substrate surface to the tallest viable tissue). Consistent with this protocol, dead apices were excluded from H measurements at DS50 after visual confirmation of tip desiccation in several pots. By contrast, *Ceratostigma minus* and *Arthraxon lanceolatus* fully recovered height after rewatering, reaching ≈ 38 cm and ≈ 44 cm, respectively—values comparable to their CK levels—indicating robust regenerative capacity. For interpretation, any DS40 “upticks” not sustained to DS50 were treated as transient growth before subsequent dieback rather than as evidence of increased drought tolerance.

LRWC, a sensitive indicator of leaf water status, declined significantly with increasing drought severity (from DS0 to DS50) but rebounded markedly following rewatering (RE10). For example, *Caryopteris forrestii* exhibited the lowest LRWC (~30%) under DS50, which increased to ~72% after rewatering; notably, *Ceratostigma minus* maintained relatively stable LRWC (ranging from 50% to 80%) across all treatments, suggesting efficient water retention capacity. LDMC generally increased under drought stress, implying that plants enhance drought resistance by allocating more dry matter to leaves: *Arthraxon lanceolatus* showed a sharp LDMC increase to ~46% under DS50 (vs. ~25% under control conditions), whereas *Cm* exhibited minor LDMC fluctuations (25–35%), indicative of a stable leaf structural profile and robust drought adaptation.

SLA decreased significantly in most species during drought (a strategy to minimize transpiration by reducing leaf area per unit dry mass). For instance, *Excoecaria acerifolia* displayed a drastic SLA reduction from ~190 cm²/g (control) to ~60 cm²/g (DS50)— 68% decrease. In contrast, *Ceratostigma minus* and *Incarvillea arguta* maintained relatively stable SLA under stress and rebounded quickly after rewatering, reflecting strong ecological plasticity and drought resilience.

### 3.3 Changes in plant physiological and biochemical indicators during drought stress rehydration process

**Figure 3:**
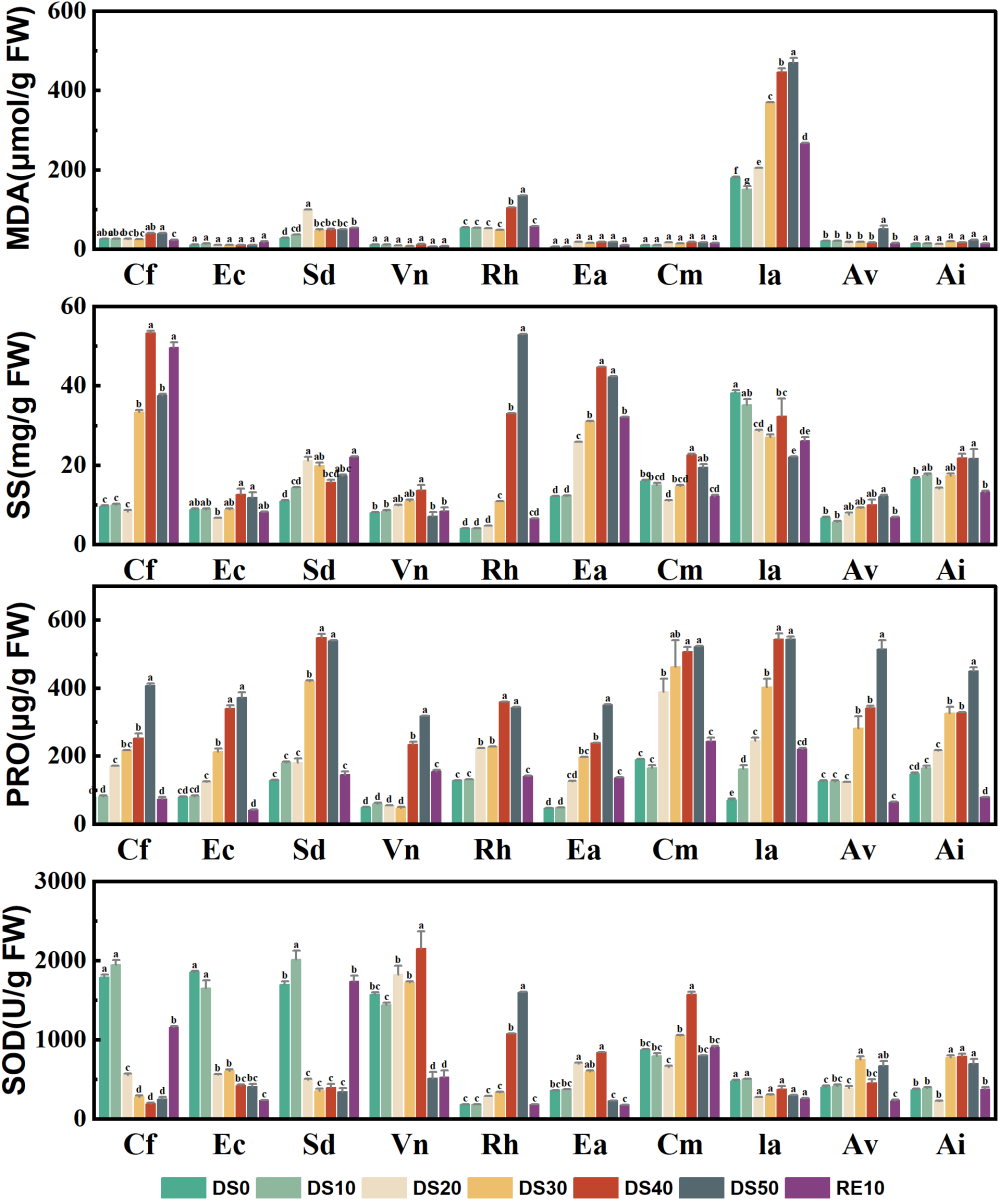
Changes in plant physiological and biochemical indicators during drought stress rehydration process. The different letters in the figure indicate statistically significant differences (P<0.05) between groups under each drought stress gradient treatment, while the same letters indicate no significant differences between groups.

**Figure 3** illustrates the changes in four key physiological and biochemical indicators—malondialdehyde (MDA), soluble sugars (SS), proline (PRO), and superoxide dismutase (SOD)—in 10 plant species under drought treatments (DS0–DS50) and rewatering (RE10). These indices serve as effective proxies for evaluating the plants’ responses to oxidative and osmotic stress, thereby indicating their drought-related physiological resilience.

MDA, a by-product of membrane lipid peroxidation, reflects the extent of oxidative damage at the cellular level. Drought stress significantly elevated MDA levels in several species. For instance, *Incarvillea arguta* exhibited a sharp increase in MDA concentration under DS50, reaching nearly 500 μmol/g FW—approximately 10 times that observed under DS0—indicating severe oxidative stress. In contrast, *Ceratostigma minus*, *Artemisia vestita*, and *Arthraxon lanceolatus* maintained relatively stable and low MDA levels under drought (mostly <80 μmol/g FW), suggesting stronger antioxidative capacity and membrane integrity. SS play a critical role in osmotic adjustment, helping cells retain water under drought. Most species showed a marked accumulation of SS under drought stress. For example, SS content in *Ia* reached ~95 mg/g FW under DS50, representing a 4.8-fold increase compared to DS0 (~20 mg/g FW). In contrast, species such as *Caryopteris forrestii*, *Elsholtzia capituligera*, and *Sophora davidii* displayed less than 2-fold increases, indicating relatively weaker osmotic regulation. Following rewatering, SS levels decreased across most species, highlighting the reversible nature of this response. PRO is another key osmolyte that accumulated broadly in response to drought. *Ia* showed the highest PRO accumulation (~680 μg/g FW) under DS50, reflecting a robust osmotic response. In contrast, *Caryopteris forrestii* and *Elsholtzia capituligera* showed only modest increases (100–300 μg/g FW), suggesting limited drought adaptability. Species such as *Ceratostigma minus* and *Arthraxon lanceolatus* maintained moderate PRO levels under drought, indicating effective but balanced osmotic regulation. SOD, a crucial antioxidant enzyme responsible for scavenging superoxide radicals, was generally upregulated during moderate drought (DS10 to DS30), but declined under severe stress (DS50) in some species—indicating that extreme drought may impair antioxidant capacity. Notably, *Ceratostigma minus* exhibited the highest SOD activity (~2800 U/g FW) under DS30, which was ~4.5 times its DS0 level, demonstrating exceptional antioxidative defense. Conversely, species such as *Vitex negundo* and *Rumex hastatus* showed consistently low SOD activity with minimal variation across treatments, suggesting limited oxidative stress response.

### 3.4 Changes in plant chlorophyll content during drought stress rehydration process

**Figure 4:**
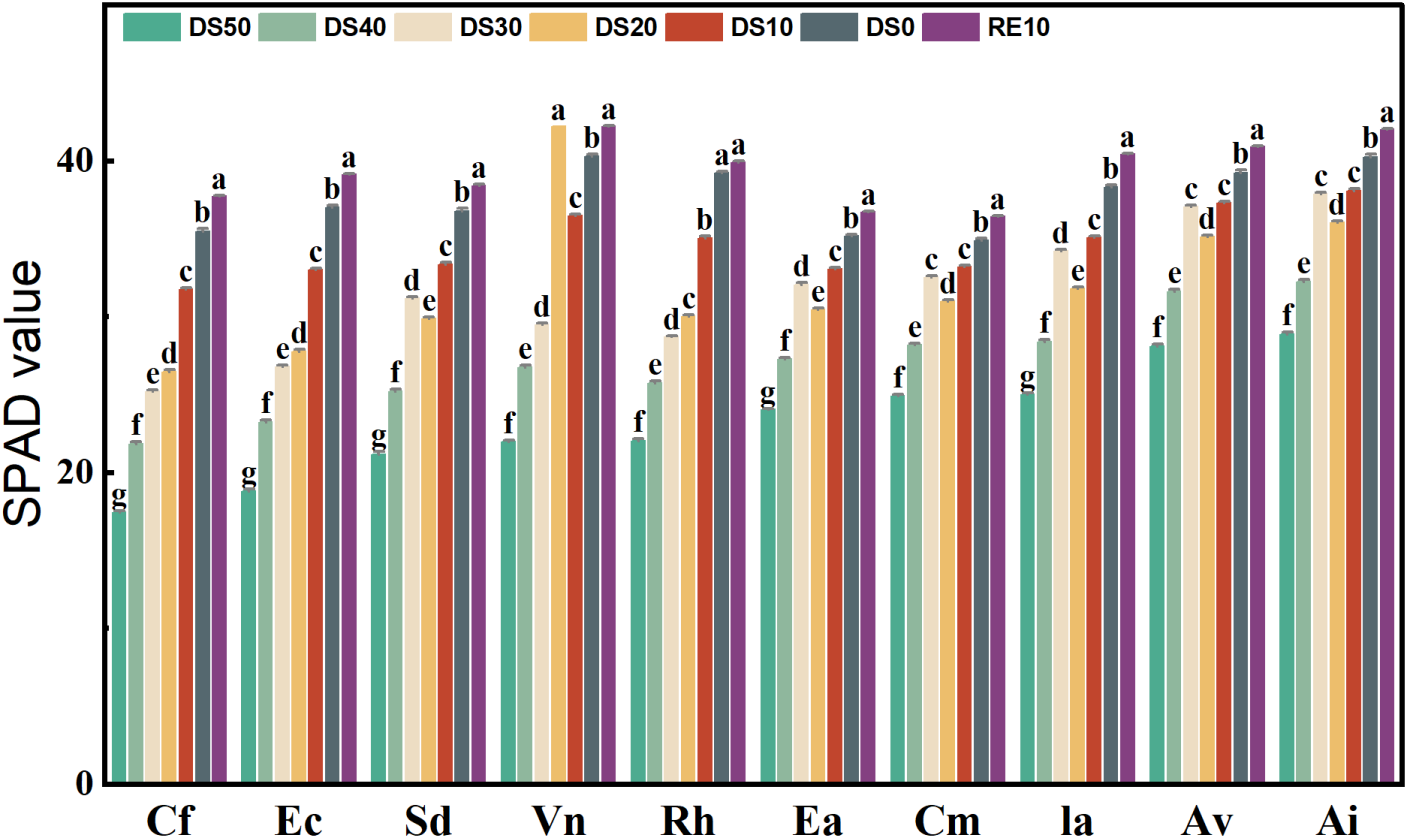
Changes in plant chlorophyll content during drought stress rehydration process. The different letters in the figure indicate statistically significant differences (P<0.05) between groups under each drought stress gradient treatment, while the same letters indicate no significant differences between groups.

**Figure 4** illustrates the variation in SPAD values (a proxy for relative chlorophyll content) across 10 plant species under different soil relative water content (SRWC) gradients. Overall, SPAD values significantly declined with increasing drought severity (DS10 → DS50) in all species (p < 0.05), indicating a strong suppression of chlorophyll synthesis under water deficit. Rewatering (RE10) led to varying degrees of SPAD recovery, although the extent of recovery was species-dependent. Under control conditions (DS0), all species maintained relatively high SPAD values, ranging from 30.56 to 45.87, representing their untreated baseline levels. As drought intensified to the most severe level (DS50), SPAD values dropped markedly. The most dramatic declines were observed in *Caryopteris forrestii*, *Elsholtzia capituligera*, and *Excoecaria acerifolia*, whose SPAD values decreased to 18.41, 20.25, and 21.08, representing reductions of 39.8%, 43.6%, and 41.7%, respectively.

In contrast, *Vitex negundo* and *Arthraxon lanceolatus* exhibited the smallest reductions under drought. Even at DS50, *Vitex negundo* and *Arthraxon lanceolatus* maintained SPAD values of 32.15 and 33.48, with only modest declines of 25.3% and 22.6% from their respective DS0 baselines—suggesting superior chlorophyll retention capacity. Following rewatering (RE10), most species showed partial recovery of SPAD values. For example, *Vitex negundo* exhibited a SPAD value of 45.02 under RE10, statistically indistinguishable from its DS0 level of 45.87 (p > 0.05), indicating a highly effective recovery of its photosynthetic system. Similarly, *Arthraxon lanceolatus* reached a SPAD value of 43.76 under RE10, nearly matching its DS0 value (43.25). Conversely, *Ec* and *Ceratostigma minus* showed relatively poor recovery, with SPAD values of only 30.13 and 29.06 under RE10—significantly lower than their DS0 baselines (37.34 and 35.92, respectively).Statistical analysis confirmed significant differences in SPAD values among treatment groups within each species (p < 0.05). In most cases, the RE10 group exhibited significantly higher SPAD values than DS40 and DS50, indicating that rewatering effectively alleviated chlorophyll suppression. However, in some species, SPAD levels under RE10 remained below control levels, suggesting that irreversible damage to the photosynthetic apparatus may have occurred under extreme drought stress.

### 3.5 Changes in photosynthetic gas exchange parameters during drought stress rehydration process

**Figure 5:**
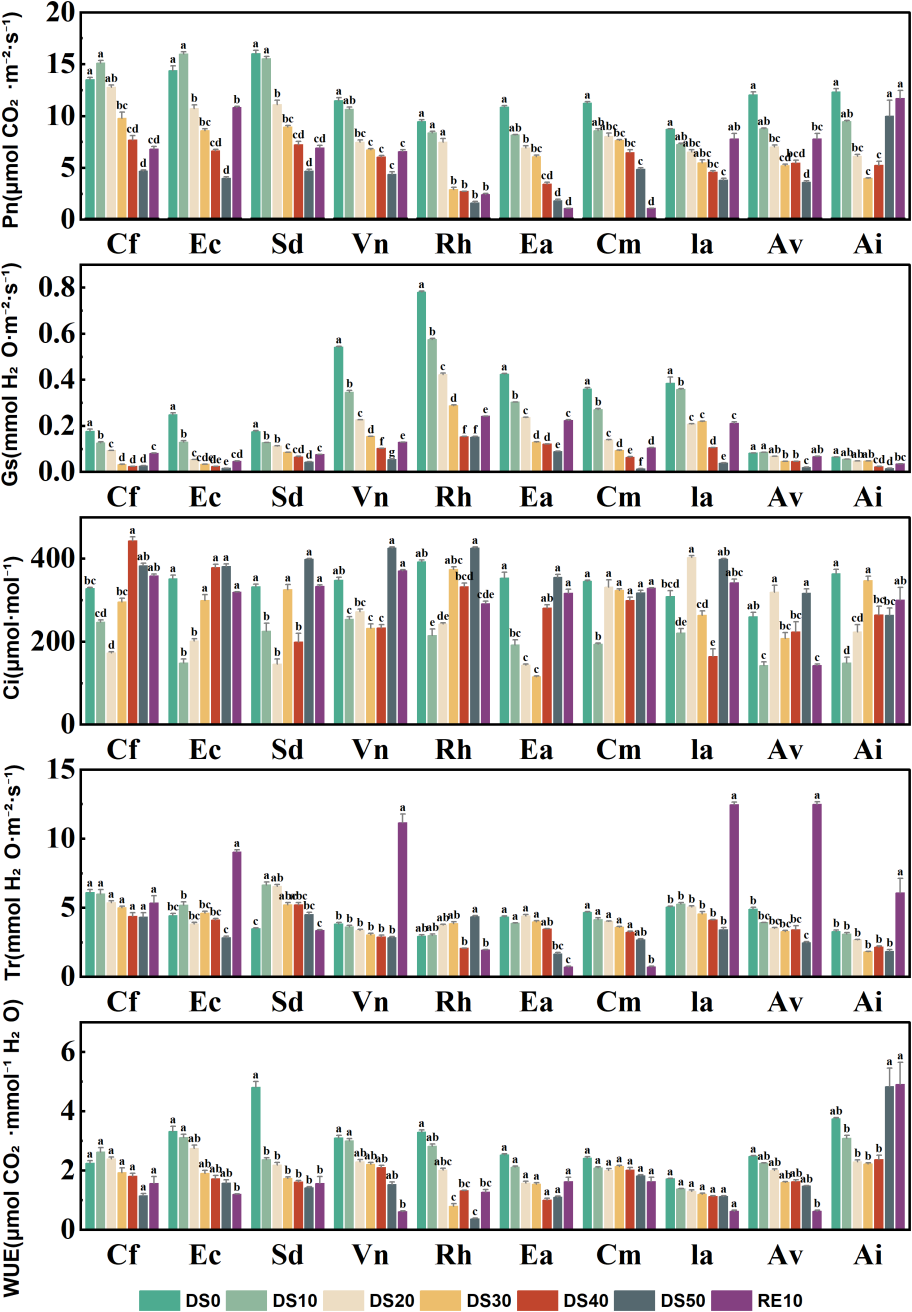
Changes in photosynthetic gas exchange parameters during drought stress rehydration process. The different letters in the figure indicate statistically significant differences (P<0.05) between groups under each drought stress gradient treatment, while the same letters indicate no significant differences between groups.

**Figure 5** illustrates the responses of five photosynthetic gas exchange parameters—net photosynthetic rate (Pn), stomatal conductance (Gs), intercellular CO_2_ concentration (Ci), transpiration rate (Tr), and water use efficiency (WUE)—in 10 plant species subjected to drought treatments (DS0–DS50) and rewatering (RE10). These parameters collectively reflect the plants’ adaptability in photosynthetic performance and stomatal regulation under drought stress. Drought stress significantly suppressed Pn in most species, with a progressive decline observed as drought severity increased (DS0 → DS50). For instance, *Caryopteris forrestii* exhibited a sharp decrease in Pn from ~15 μmol·m^-2^·s^-1^ under DS0 to ~3 μmol·m^-2^·s^-1^ under DS50—an 80% reduction. In contrast, *Incarvillea arguta* and *Ceratostigma minus* maintained relatively higher Pn values (~7–10 μmol·m^-2^·s^-1^) even under DS30, indicating that their photosynthetic capacity was less impaired by drought. After rewatering (RE10), Pn values recovered significantly in most species, particularly in *Ceratostigma minus* and *Incarvillea arguta*, both of which nearly returned to their DS0 levels, suggesting strong resilience in photosynthetic function.Stomatal conductance (Gs), a key indicator of stomatal aperture, showed a universal decline under drought, reflecting an adaptive closure of stomata to reduce water loss. *Vitex negundo* had the highest Gs (0.8 mol·m^-2^·s^-1^) under DS0 but dropped below 0.05 mol·m^-2^·s^-1^ under DS50. In contrast, *Ceratostigma minus* and *Arthraxon lanceolatus* exhibited a slower decline in Gs under drought, suggesting greater capacity to maintain gas exchange through more controlled stomatal regulation. Intercellular CO_2_ concentration (Ci) either slightly declined or remained stable in most species as drought intensified, implying that the reduction in Pn was predominantly driven by non-stomatal limitations. For example, *Ceratostigma minus* and *Incarvillea arguta* maintained relatively stable Ci levels (220–280 μmol·mol^-1^) across drought treatments, whereas *Caryopteris forrestii* and *Vitex negundo* showed significant declines in Ci to below 180 μmol·mol^-1^ under severe drought, indicative of compromised photosynthetic capacity.Transpiration rate (Tr) decreased markedly under drought stress, with most species registering Tr values below 1 mmol·m^-2^·s^-1^ under DS50. *Ceratostigma minus*, however, sustained a relatively high Tr (~2 mmol·m^-2^·s^-1^) even under DS50, compared to ~4.5 mmol·m^-2^·s^-1^ under DS0, highlighting its stronger water regulation capacity. Conversely, *Caryopteris forrestii*, *Vitex negundo*, and *Rumex hastatus* exhibited the steepest declines in Tr, reflecting more aggressive transpiration suppression, which, while conserving water, may also compromise Pn.Water use efficiency (WUE), which integrates carbon assimilation and water loss, varied substantially among species. Some species demonstrated significantly elevated WUE under drought. For example, *Arthraxon lanceolatus* reached a peak WUE of 6.5 μmol CO_2_·mmol^-1^ H_2_O under DS30 and DS40, compared to ~2.8 under DS0. In contrast, *Vitex negundo* and *Elsholtzia capituligera* exhibited limited improvement in WUE during drought, suggesting a weaker coordination between photosynthesis and transpiration. Following rewatering, WUE values generally returned to intermediate levels, although species such as *Ceratostigma minus*, *Incarvillea arguta*, and *Arthraxon lanceolatus* maintained relatively high WUE, reflecting sustained physiological efficiency.

### 3.6 Changes in chlorophyll fluorescence parameters during drought stress rehydration process

**Figure 6:**
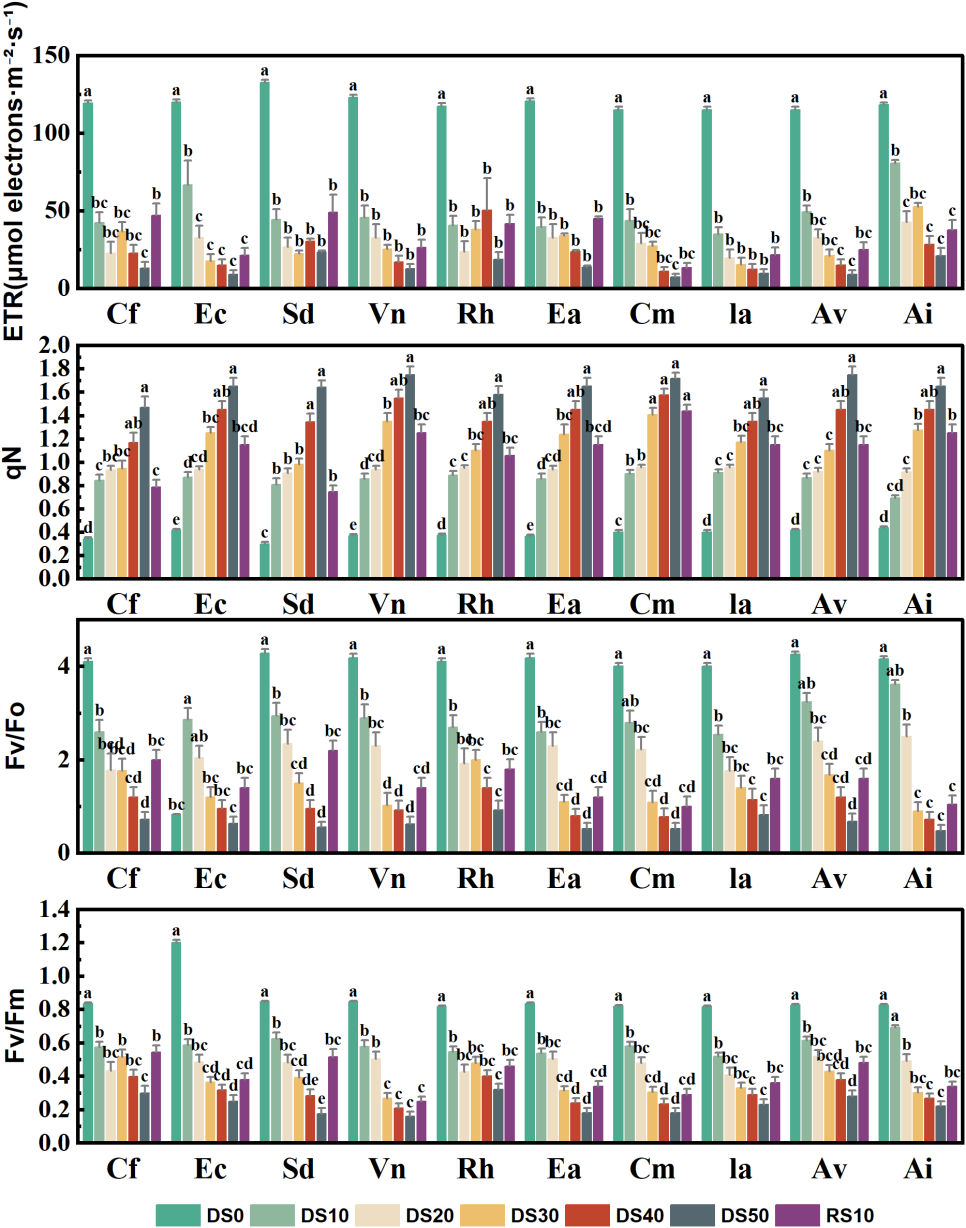
Changes in chlorophyll fluorescence parameters during drought stress rehydration process. The different letters in the figure indicate statistically significant differences (P<0.05) between groups under each drought stress gradient treatment, while the same letters indicate no significant differences between groups.

**Figure 6** presents the variation in four key chlorophyll fluorescence parameters—electron transport rate (ETR), non-photochemical quenching (qN), the maximum initial fluorescence ratio (Fv/F_0_), and maximum quantum efficiency of PSII (Fv/Fm)—for 10 plant species subjected to varying levels of drought stress (DS0–DS50) and rewatering (RE10). These parameters are critical indicators of Photosystem II (PSII) functionality under abiotic stress, providing insight into photoinhibition, energy dissipation, and overall drought tolerance. Drought stress significantly reduced ETR across all species, indicating impaired electron flow and a decline in photosynthetic performance. Most species showed ETR values below 40 under DS50. For example, *Cf* exhibited a sharp drop from ~130 under DS0 to ~30 under DS50, representing a >75% reduction. In contrast, *Ceratostigma minus*, *Incarvillea arguta*, and *Arthraxon lanceolatus* maintained ETR values in the range of 50–60 under DS50 and showed substantial recovery following rewatering, suggesting a more stable PSII performance and enhanced drought resilience.Non-photochemical quenching (qN), an indicator of excess energy dissipation mechanisms, generally increased under drought, reflecting enhanced photoprotective responses. For instance, *Incarvillea arguta* and *Arthraxon lanceolatus* exhibited pronounced increases in qN values under DS30 to DS50, reaching as high as 1.6–1.8—more than double their baseline levels under DS0 (~0.6). Conversely, *Caryopteris forrestii* and *Elsholtzia capituligera* showed only marginal increases in qN, implying weaker thermal dissipation capacity and potentially greater susceptibility to photoinhibition. After rewatering, qN values decreased across most species but remained above DS0 levels, indicating possible physiological memory or incomplete recovery of energy dissipation pathways. Fv/F_0_, which reflects PSII structural integrity, declined markedly under drought, highlighting increasing damage to the photosynthetic apparatus. For example, *Caryopteris forrestii* and *Vitex negundo* exhibited a reduction from 3.5–3.8 under DS0 to ~1.0 under DS50—over a 70% decrease. In contrast, *Ceratostigma minus*, *Incarvillea arguta*, and *Arthraxon lanceolatus* retained Fv/F_0_ values between 1.8 and 2.2 under DS50, suggesting less severe structural damage and improved capacity for recovery. Fv/Fm, a widely accepted indicator of the maximum quantum efficiency of PSII photochemistry (typically ~0.8 in healthy plants), also declined significantly under drought. In severely stressed plants such as *Caryopteris forrestii*, *Elsholtzia capituligera*, and *Vitex negundo*, Fv/Fm fell below 0.4 under DS50, indicating substantial impairment of PSII photochemistry. Conversely, *Ceratostigma minus* and *Arthraxon lanceolatus* showed relatively moderate declines, maintaining Fv/Fm values above 0.6 under drought and recovering to >0.75 following rewatering, reflecting strong PSII resilience and repair capacity.

### 3.7 Changes in plant nitrogen and phosphorus utilization efficiency during drought stress rehydration process

**Figure 7:**
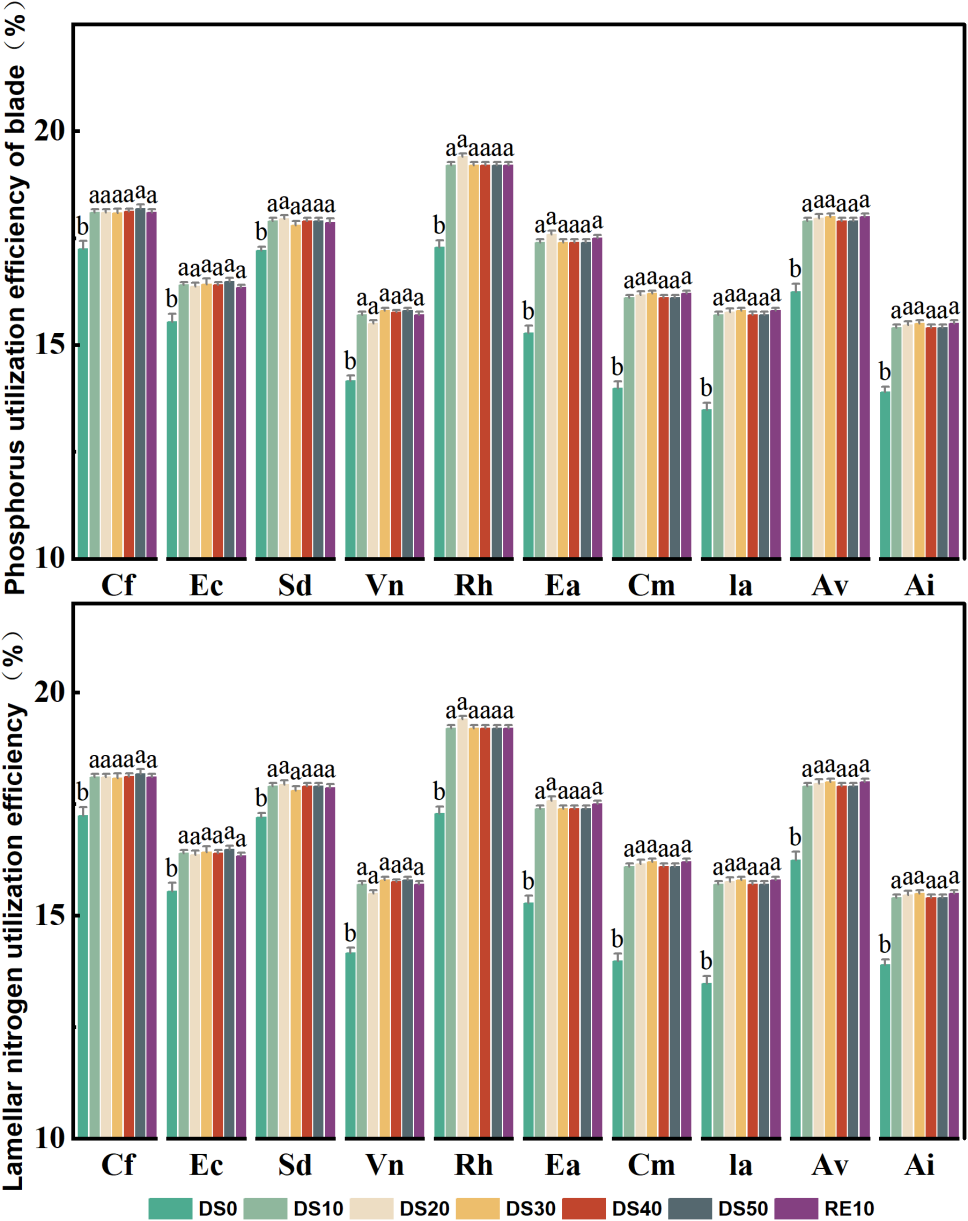
Changes in plant nitrogen and phosphorus utilization efficiency during drought stress rehydration process. The different letters in the figure indicate statistically significant differences (P<0.05) between groups under each drought stress gradient treatment, while the same letters indicate no significant differences between groups.

**Figure 7** illustrates the variation in phosphorus use efficiency (PUE) and nitrogen use efficiency (NUE) in the leaves of 10 plant species subjected to drought stress (DS0–DS50) and subsequent rewatering (RE10). These two parameters are critical indicators of a plant’s ability to optimize nutrient allocation under resource-limited conditions, serving as important physiological metrics for drought tolerance evaluation. Under drought stress, most species exhibited a general increasing trend in PUE, particularly during mild to moderate drought stages (DS10–DS30), indicating that plants enhance the photosynthetic efficiency per unit phosphorus as a drought adaptation strategy. *Rumex hastatus* and *Excoecaria acerifolia* showed the highest PUE values under DS20 and DS30, reaching ~20%, which represents an increase of approximately 3–5 percentage points compared to DS0. In contrast, *Caryopteris forrestii* and *Vitex negundo* displayed smaller increases in PUE (remaining within 14–16%), suggesting limited capacity for phosphorus utilization enhancement under stress. *Ceratostigma minus* and *Arthraxon lanceolatus* maintained relatively stable PUE values (16–18%) across all treatments, highlighting their capacity for phosphorus homeostasis and efficient nutrient regulation. Following rewatering (RE10), PUE values in most species returned to DS0 levels or slightly declined, suggesting that phosphorus utilization mechanisms are partially reversible after stress release.The response pattern of NUE to drought was largely consistent with that of PUE. For most species, NUE increased significantly under moderate drought, reflecting improved nitrogen allocation efficiency under limited nitrogen availability. *Rumex hastatus* and *Excoecaria acerifolia* reached peak NUE values (~20%) under DS20 and DS30, indicating superior nitrogen utilization under drought conditions. *Caryopteris forrestii*, *Elsholtzia capituligera*, and *Vitex negundo* exhibited relatively minor changes in NUE, fluctuating between 15–17% throughout the treatment period, implying a limited capacity for nitrogen-use optimization. In contrast, *Ceratostigma minus* and *Arthraxon lanceolatus* maintained stable NUE values (17–19%) regardless of drought or rewatering conditions, suggesting that their nitrogen uptake and assimilation systems are both robust and adaptive. After rewatering (RE10), NUE values in most species decreased to levels similar to DS0, indicating that drought-induced nitrogen regulation strategies are both plastic and reversible.

### 3.8 Changes in plant hydraulic properties during drought stress rehydration process

**Figure 8:**
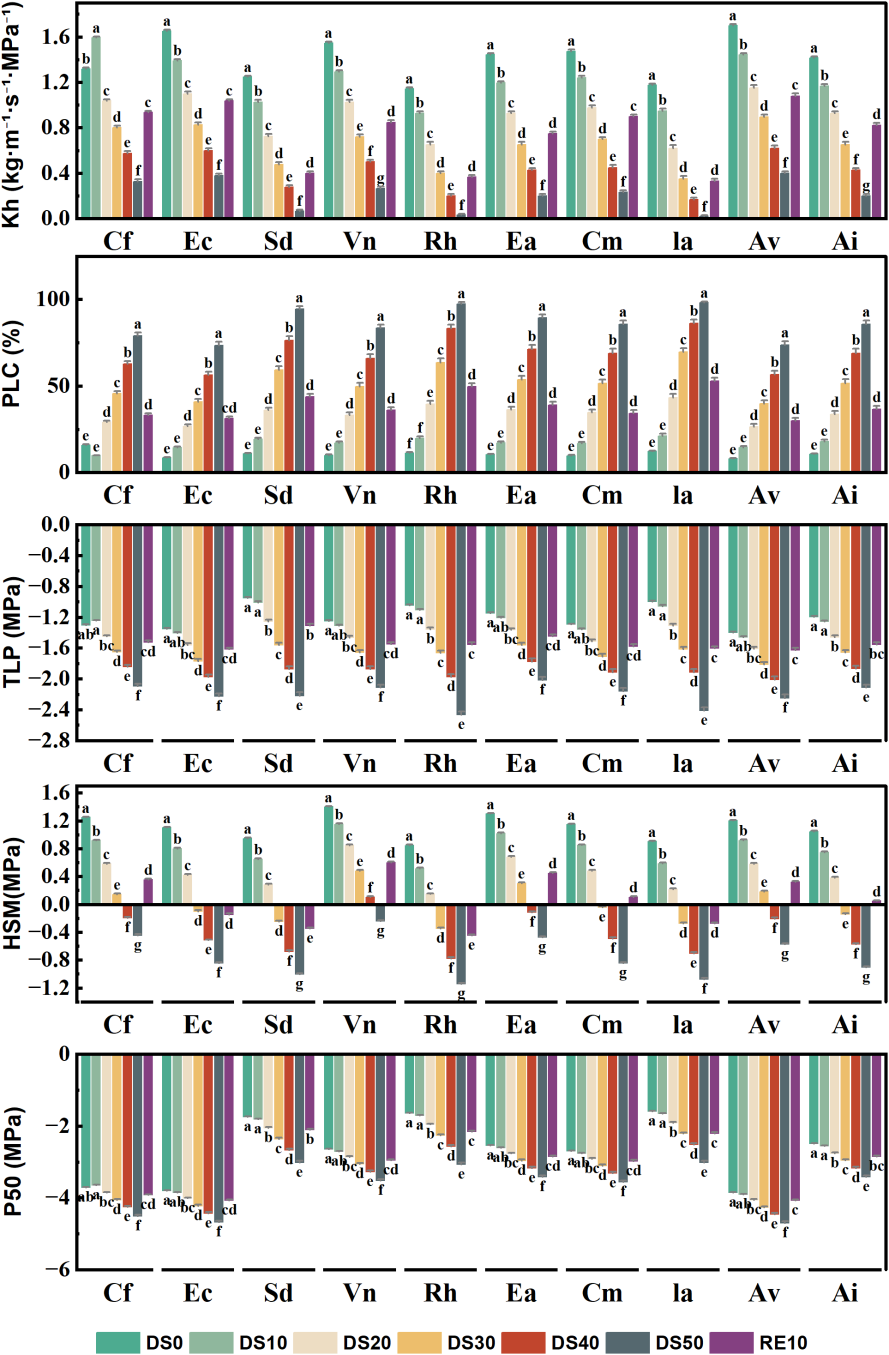
Changes in plant hydraulic properties during drought stress rehydration process. The different letters in the figure indicate statistically significant differences (P<0.05) between groups under each drought stress gradient treatment, while the same letters indicate no significant differences between groups.

**Figure 8** presents the variation in five key hydraulic traits across 10 plant species subjected to different drought treatments (DS0–DS50) and rewatering (RE10), including xylem hydraulic conductivity (Kh), percentage loss of conductivity (PLC), turgor loss point (TLP), water potential sensitivity index (|dΨ/dM|), and the water potential at 50% loss of conductivity (P50). Together, these traits comprehensively reflect the stability of the hydraulic system, resistance to embolism, and hydraulic adjustment strategies under drought stress.Kh significantly declined with increasing drought severity, indicating reduced water transport capacity. For example, *Caryopteris forrestii* showed a drastic reduction in Kh from approximately 0.78 under DS0 to 0.15 under DS50 (a decline of ~80%). In contrast, *Ceratostigma minus* and *Incarvillea arguta* maintained Kh values between 0.35 and 0.45 under DS50, suggesting more stable hydraulic conductance. Following rewatering (RE10), Kh partially recovered in most species, though recovery extent varied considerably among species. PLC quantifies the degree of xylem embolism, with higher values indicating greater hydraulic failure. Most species exhibited sharp increases in PLC under DS50. For instance, *Sophora davidii* showed an increase from ~12% under DS0 to ~65% under DS50. However, *Ceratostigma minus* and *Arthraxon lanceolatus* maintained lower PLC values (~45%) even under severe drought, indicating stronger embolism resistance. After rewatering, PLC values generally declined, indicating partial reversibility of xylem dysfunction. TLP, which reflects the water potential at which leaf cells lose turgor, shifted towards more negative values in all species under drought stress, indicating enhanced osmotic adjustment. For example, *Rumex hastatus* exhibited a decrease in TLP from –0.8 MPa (DS0) to –1.45 MPa (DS50), while *Ceratostigma minus* and *Incarvillea arguta* only decreased to ~–1.1 MPa, suggesting better cellular turgor maintenance under drought. The hydraulic safety margin (HSM), representing the gap between the minimum midday stem water potential and P50, reflects the sensitivity of plant water status to drought. A larger HSM indicates a higher vulnerability. Under drought conditions, most species showed a marked increase in HSM. For instance, *Elsholtzia capituligera* increased from ~0.1 (DS0) to 0.5 (DS50). In contrast, species such as *Ceratostigma minus*, *Artemisia vestita*, and *Arthraxon lanceolatus* maintained lower HSM values (<0.3) throughout, indicating more conservative water use strategies and greater drought resilience.P50, the water potential at which 50% of xylem conductivity is lost, is a critical index of drought-induced embolism resistance. Species with more negative P50 values are considered more drought-tolerant. Notable interspecific variation was observed: *Caryopteris forrestii*, *Elsholtzia capituligera*, and *Sophora davidii* had P50 values around –1.5 MPa under DS50, whereas *Ceratostigma minus* and *Arthraxon lanceolatus* reached more negative values of –2.2 MPa and –2.5 MPa, respectively, demonstrating superior hydraulic safety. After rewatering, P50 values partially recovered but remained lower than DS0 levels, suggesting that drought-induced xylem damage may be partially irreversible.

### 3.9 Correlation between hydraulic and other characteristics

**Figure 9:**
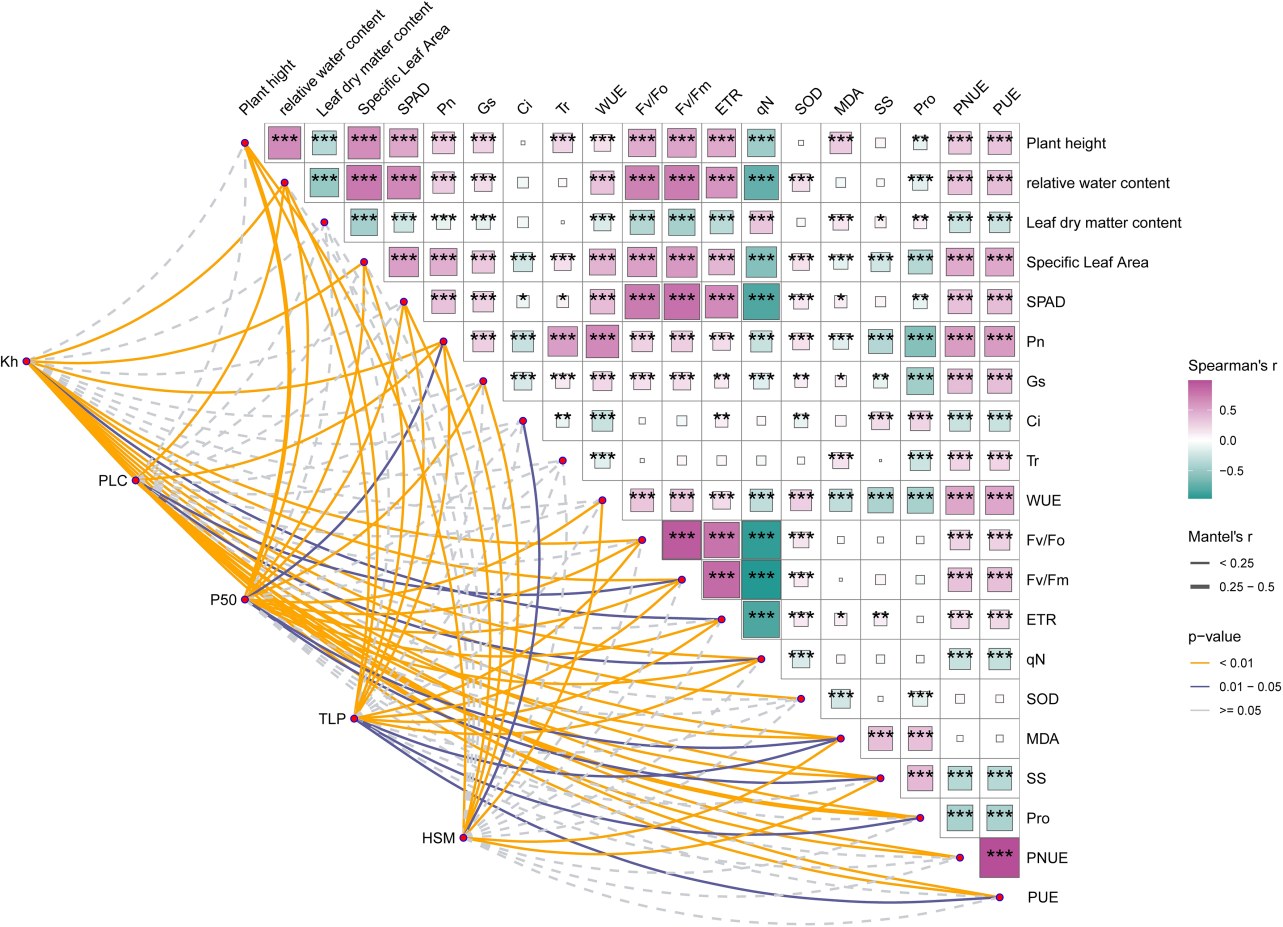
Correlation between hydraulic and other characteristics. The symbols *, **, and *** indicate different levels of statistical significance between groups, where * denotes p < 0.05, ** denotes p < 0.01, and *** denotes p < 0.001.

As shown in **Figure 9**, To quantify cross-trait coordination, we analyzed 25 drought-related indicators using Spearman rank correlations and Mantel tests. For interpretability, hydraulic thresholds are discussed with the usual convention that more negative raw P50/TLP denote greater safety; where noted, effect directions reflect that convention.

Plant height (PH) showed broad, significant positive associations (P < 0.01) with relative water content (RWC), specific leaf area (SLA), net photosynthetic rate (Pn), stomatal conductance (Gs), water-use efficiency (WUE), hydraulic conductivity (Kh), embolism resistance (more negative P50), turgor loss point (TLP; more negative), PSII metrics (Fv/Fo, Fv/Fm), electron transport rate (ETR), SPAD chlorophyll, and nutrient-use efficiencies (PNUE, PUE). PH correlated negatively with leaf dry-matter content (LDMC), percent loss of conductivity (PLC), proline (Pro), and non-photochemical quenching (qN) (P < 0.01). RWC mirrored this pattern—positive with SLA, Pn, Gs, WUE, Kh, (more negative) P50 and TLP, PNUE, PUE, Fv/Fo, Fv/Fm, ETR, SPAD, and negative with LDMC, PLC, soluble sugars (SS), Pro, and qN (P < 0.01)—underscoring water status as a nexus for carbon gain and hydraulic function.

As a structural proxy, LDMC was positively related to PLC, SS, Pro, and qN (P < 0.01), but negatively to SLA, Pn, Gs, Kh, Fv/Fo, Fv/Fm, ETR, and SPAD—consistent with denser leaves coinciding with reduced photosynthetic efficiency and sap flux capacity. By contrast, SLA aligned positively with Pn, Gs, transpiration (Tr), WUE, Kh, (more negative) P50 and TLP, PNUE, PUE, Fv/Fo, Fv/Fm, ETR, and SPAD, and negatively with PLC, SS, Pro, and qN (P < 0.01), indicating that structurally efficient leaves tended to sustain both higher photochemical performance and water transport.

Pn correlated positively with Gs, Tr, WUE, Kh, (more negative) TLP, PNUE, PUE, Fv/Fo, Fv/Fm, ETR, and SPAD, but negatively with intercellular CO_2_ (Ci), PLC, SS, Pro, and qN. These associations reinforce that drought-related Pn declines track transport limitation (lower Kh, higher PLC) and stress metabolite accumulation (SS, Pro), alongside increased energy dissipation (qN).

Within the hydraulic block, Kh and P50 (more negative = safer) were strongly connected to multiple photosynthetic and antioxidative traits. Mantel tests yielded r > 0.25 between the hydraulic trait set and several photochemical/antioxidant parameters, indicating that hydraulic safety underpins carbon assimilation while coordinating photochemical stability and non-structural metabolism. Network polarity was clear: Kh, (more negative) P50, and (more negative) TLP acted as pivotal hubs linking growth, water status, and photosynthesis, whereas PLC and qN formed a negative-response core associated with embolism formation and heightened thermal dissipation under severe stress.

Together, these results support a coordinated hydraulic–photosynthetic–metabolic regulation of drought response: species (and treatments) that maintain hydraulic integrity preserve water status and photochemistry, mobilize antioxidants efficiently, and minimize costly osmotic/photoprotective escalation, whereas systems trending toward high PLC and qN enter failure-prone domains. This multiscale integration and trait trade-off framework clarifies why hydraulic metrics are powerful predictors in comprehensive drought-tolerance assessments.

**Figure 10:**
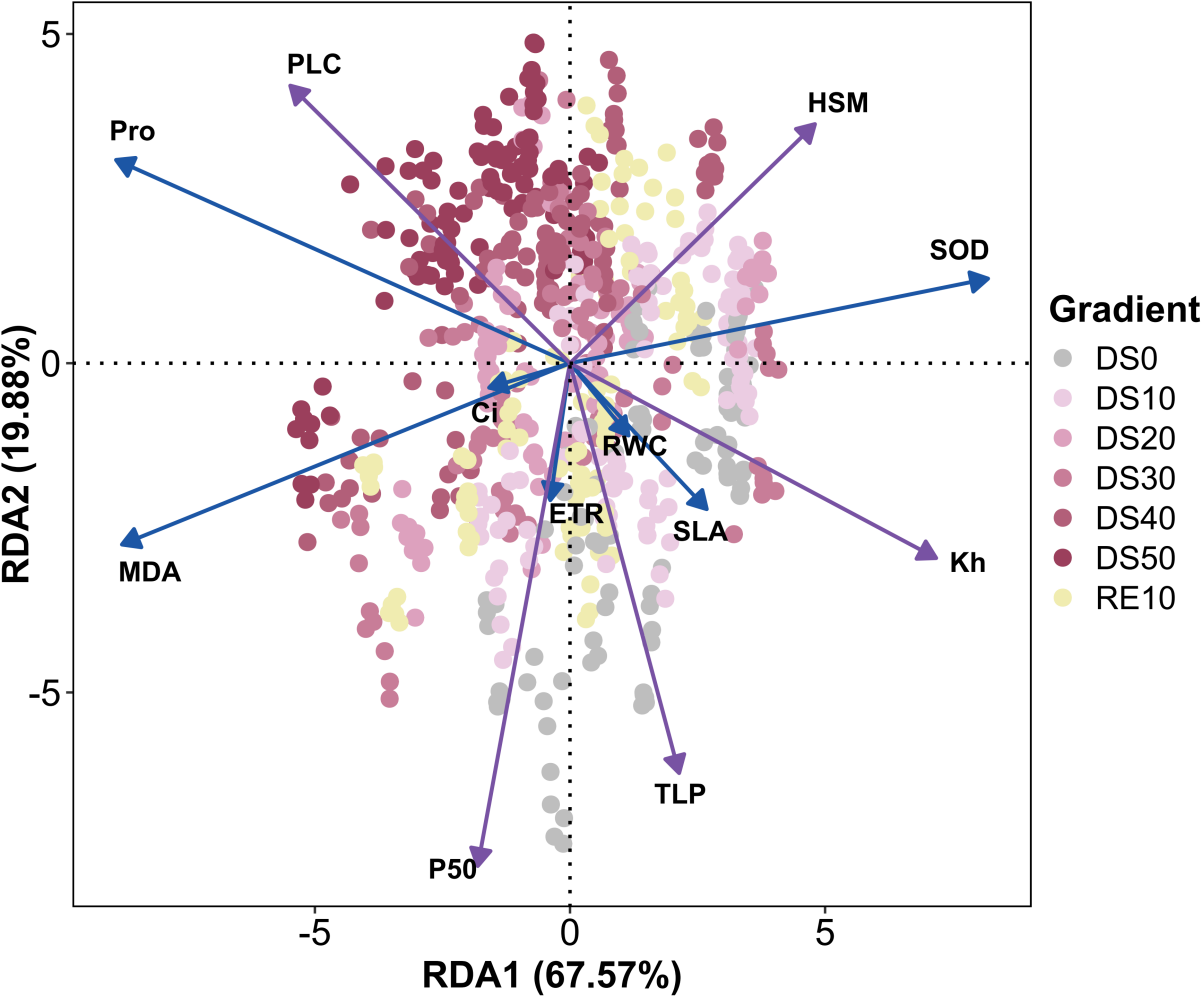
Redundancy analysis of indicators during drought stress rehydration process. The complete names of the indicator variables presented in the figure are as follows: ETR (Electron Transport Rate), Fv/Fm (Maximum Photochemical Efficiency), Fv/Fo (Maximum Initial Fluorescence Ratio), SPAD (Soil Plant Analysis Development Value), RWC (Relative Water Content), SLA (Specific Leaf Area), Pn (Net Photosynthetic Rate), Gs (Stomatal Conductance), TLP (Turgor Loss Point), PUE (Phosphorus Use Efficiency), PNUE (Nitrogen Use Efficiency), WUE (Water Use Efficiency), Kh (Hydraulic Conductivity), P50 (Water Potential at 50% Loss of Conductivity), HSM (Hydraulic Safety Margin), LDMC (Leaf Dry Matter Content), PLC (Percentage Loss of Conductivity), SS (Soluble Sugar), MDA (Malondialdehyde), Ci (Intercellular CO_2_ Concentration), Pro (Proline), and qN (Non-Photochemical Quenching Coefficient).

As shown in **Figure 10**, To resolve how hydraulics structure whole‐plant drought responses, we performed redundancy analysis (RDA) using five hydraulic variables (P50, Kh, TLP, HSM, PLC) as constraining factors and a suite of drought-responsive traits (ETR, RWC, SLA, Ci, MDA, Pro, SOD) as responses. The first two canonical axes explained 87.45% of the constrained variance (RDA1 67.57%, RDA2 19.88%), indicating that hydraulic status exerts strong control over physiological and ecological performance across the drought–rewatering trajectory. Vector orientations on the biplot (sign-standardized so that more negative P50 and TLP map in the direction of greater safety) showed Kh, TLP, HSM, and P50 aligned positively along RDA1 and closely colinear with ETR, RWC, SLA, and SOD. This configuration identifies RDA1 as a gradient from hydraulic integrity to failure: species or treatments with stable transport capacity and more negative turgor thresholds sustained higher leaf water content, preserved photochemistry, and mobilized antioxidative defenses—collectively maintaining photosynthetic function under deficit. In contrast, PLC, MDA, and Pro loaded negatively on RDA1 and clustered with severe-drought samples (DS40, DS50), capturing a syndrome of embolism formation, oxidative damage, and osmotic stress signaling that typifies failure domains. Treatment centroids corroborated this interpretation: DS10/DS20 and RE10 occupied the positive side of RDA1, co-occurring with Kh, SOD, RWC, and ETR, consistent with higher resilience under moderate stress and accelerated functional recovery after rewatering. By comparison, severe-stress groups shifted toward the PLC–MDA–Pro sector. Ci plotted near the origin, implying limited discriminatory leverage when hydraulics dominate variation (stomatal and biochemical controls act in opposing directions). The close directional alignment of SLA with TLP further supports a coordinated adjustment between leaf structural traits and cellular water-relation thresholds. In sum, the RDA demonstrates that hydraulic traits—particularly Kh, P50, and TLP—are central nodes that not only delineate physical tolerance limits but also govern photosynthetic performance, antioxidative capacity, and osmotic regulation. These results provide a robust mechanistic basis for hydraulic trait–based screening of drought-resilient species and justify their inclusion in minimal diagnostic panels for restoration in dry–hot valleys.

### 3.10 Comprehensive evaluation of plant drought resistance

**Figure 11:**
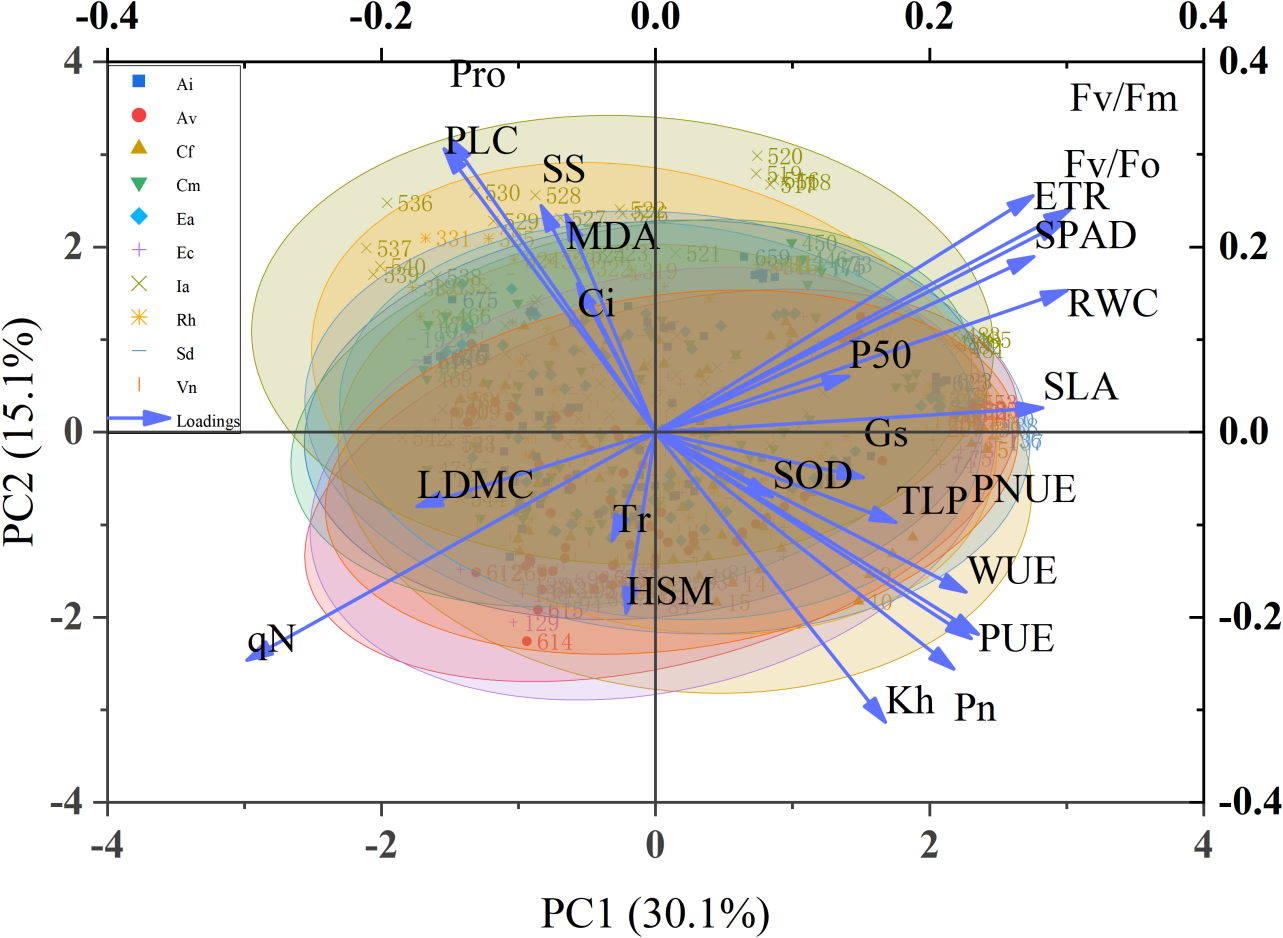
Principal component analysis of indicators during drought stress rehydration process.

As shown in **Figure 11**, To integrate multi-trait responses across the drought–rewatering cycle, we performed principal component analysis (PCA) on hydraulic, photosynthetic, water-status, and biochemical variables for the ten species. The first two components explained 45.2% of the variance (PC1 = 30.1%, PC2 = 15.1%). We oriented component signs such that higher PC1 scores reflect better water/carbon status and photochemical performance.

On the biplot, key indicators of functional performance—water-use efficiency (WUE), stomatal conductance (Gs), net photosynthetic rate (Pn), relative water content (RWC), maximum photochemical efficiency (Fv/Fm), electron transport rate (ETR), and SPAD—loaded strongly and positively on PC1, defining a common axis of photosynthetic capacity and water retention. Hydraulic traits indexing transport capacity and safety (greater Kh; more negative P50; more negative TLP, expressed with sign alignment for interpretability) and antioxidant potential (SOD) projected in the same quadrant, indicating synergy among water transport, photochemistry, and ROS detoxification along the primary resistance axis. By contrast, stress-response markers—malondialdehyde (MDA), soluble sugars (SS), and proline (Pro)—extended chiefly along PC2, capturing the intensity of osmotic/oxidative response rather than steady-state efficiency.

Drought-tolerant taxa—*Caryopteris forrestii*, *Sophora davidii*, *Rumex hastatus*, and *Elsholtzia capituligera*—clustered at higher PC1 scores, combining superior hydraulic status (wider HSM via more negative P50/TLP and higher Kh) with stronger photochemical performance (higher Fv/Fm, ETR) and elevated WUE. In contrast, drought-sensitive species—*Ceratostigma minus*, *Incarvillea arguta*, *Artemisia vestita*, and *Arthraxon lanceolatus*—occupied lower PC1 regions and were associated with higher qN, intercellular CO_2_ concentration (Ci), and leaf dry-matter content (LDMC), a constellation indicative of photoprotective energy dissipation under stress, low water-use economy, structural densification, and incipient hydraulic limitation. Consistent with these patterns, percent loss of conductivity (PLC) opposed Pn, Gs, and Kh, reinforcing the view that embolism formation delineates failure domains on PC1.

Taken together, the PCA clarifies that drought performance is governed by coordinated hydraulic–physiological adjustment: a primary axis (PC1) linking hydraulic safety/efficiency to photosynthetic function and water status, and a secondary axis (PC2) reflecting the magnitude of osmotic and oxidative responses following stress. This multivariate structure justifies our use of weighted PCA and fuzzy-membership scoring in the comprehensive ranking, minimizing bias from any single trait and yielding a stable, quantitative basis for cross-species comparisons.

Based on the principal component analysis (PCA) of 25 physiological and biochemical drought-related traits—encompassing gas exchange parameters, chlorophyll fluorescence indicators, and biochemical indices—a set of initial eigenvalues, explained variances, and cumulative rotated loadings were obtained (**Table 2**). The results show that the first seven principal components accounted for 96.971% of the total variance, indicating that these components sufficiently represent the information contained in all 25 drought-resistance indicators. Therefore, the first seven principal components (Y_1_–Y_7_) were calculated using the standardized dataset, the factor score coefficient matrix, and the rotated component loadings. The proportion of each eigenvalue relative to the sum of all extracted eigenvalues was used as the weight to compute a comprehensive drought-resistance score for each of the seven studied species. This score provides a robust, integrative index for evaluating plant adaptability to drought stress within the study region.

**Table 2:** Principal component analysis results of plant indicators.

Component	Initial eigenvalues		
	Total	% of variance	Cumulative %
1	8.603	34.411	34.411
2	5.287	21.146	55.557
3	3.951	15.803	71.36
4	2.362	9.446	80.806
5	1.586	6.343	87.149
6	1.373	5.49	92.64
7	1.083	4.331	96.971

According to the comprehensive evaluation based on the scores of the seven principal components and the membership function analysis (**Table 3**), the ranking of drought resistance among the ten plant species from strongest to weakest is as follows: *Rumex hastatus* > *Caryopteris forrestii* > *Sophora davidii* > *Vitex negundo* >*Elsholtzia capituligera* > *Artemisia vestita* > *Arthraxon lanceolatus* > *Incarvillea arguta* > *Ceratostigma minus* > *Excoecaria acerifolia*.

**Table 3:** Comprehensive evaluation results of plant drought resistance.

Plant species	Y1	Y2	Y3	Y4	Y5	Y6	Y7	Comprehensive score	Sorting
Cf	1.655	-0.115	1.644	-0.355	-0.152	-0.273	1.121	3.526	2
Ec	0.416	-0.561	-0.554	0.705	-1.008	0.455	0.223	-0.323	5
Sd	-0.315	-0.271	-0.259	-0.142	2.034	-0.417	1.376	2.006	3
Vn	-0.047	-0.669	-1.271	-0.524	-0.195	1.942	0.560	-0.204	4
Rh	-0.313	2.061	0.751	1.115	0.470	1.232	-0.448	4.868	1
Ea	0.217	0.270	0.368	-2.219	-0.129	0.149	-1.383	-2.728	10
Cm	-0.774	1.398	-1.068	-0.495	-1.005	-1.454	0.810	-2.587	9
Ia	-2.020	-1.202	1.393	0.317	-0.634	-0.156	-0.087	-2.390	8
Av	1.072	-0.369	-0.394	1.182	-0.677	-0.730	-0.665	-0.581	6
Ai	0.109	-0.542	-0.610	0.415	1.296	-0.749	-1.506	-1.586	7

**Figure 12:**
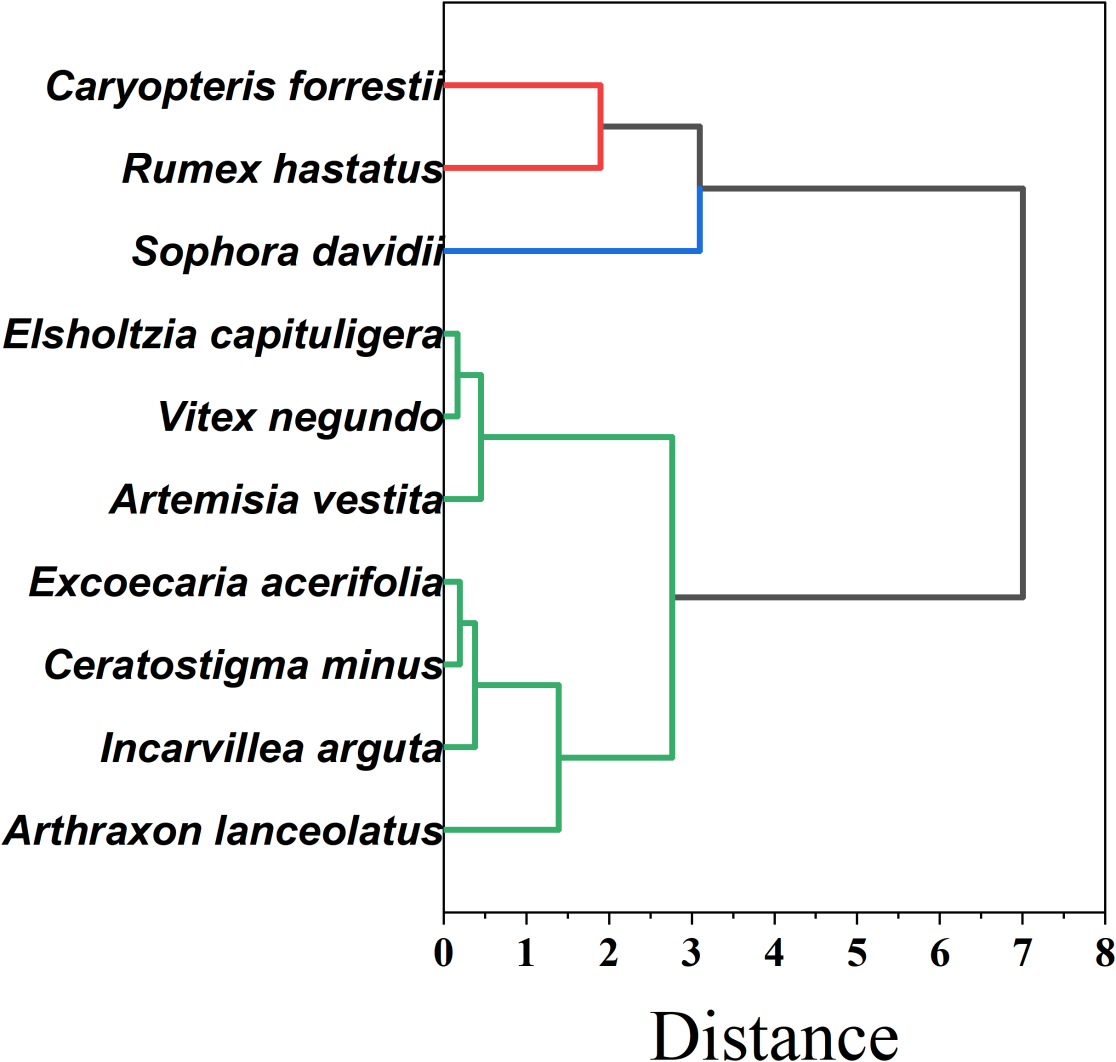
Cluster analysis results of plant drought resistance.

As shown in **Figure 12**, To further elucidate the overall similarity and functional differentiation patterns of drought resistance among the ten plant species, a cluster analysis was conducted based on 25 drought-related traits spanning morphological, photosynthetic, hydraulic, physiological, and nutrient-use dimensions. Standardized data were used to construct a Euclidean distance matrix, and Ward’s minimum variance method was applied for hierarchical clustering. The results clearly grouped the ten species into three distinct clusters, indicating a well-defined pattern of functional divergence.

Group I — Hydraulic-safe & physiologically resilient (*Caryopteris forrestii*, *Rumex hastatus*, *Sophora davidii*).These species combine more negative P50 and wide hydraulic safety margins (HSM) with robust photochemical performance (high Fv/Fm) and resource-use efficiencies (WUE, PNUE). The trait constellation indicates strong water-retention capacity and sustained carbon assimilation under prolonged drought—i.e., a conservative, safety-first strategy well suited to the driest microsites. Group II — Fast-growing, photosynthesis-driven (*Elsholtzia capituligera*, *Vitex negundo*, *Artemisia vestita*).Characterized by high SLA and elevated Pn/ETR, these taxa capitalize on wet pulses, but less negative P50 and narrower HSM signal weaker hydraulic tolerance during severe or long droughts. This acquisitive strategy favors rapid resource capture in seasons or sites with controllable water availability. Group III — Hydraulically and physiologically vulnerable (*Excoecaria acerifolia*, *Ceratostigma minus*, *Incarvillea arguta*, *Arthraxon lanceolatus*). Lower values across key indices (e.g., Kh, WUE, SOD, PNUE) and limited post-stress recovery indicate higher drought sensitivity and dependence on favorable moisture. These species warrant cautious deployment and, where used, should be matched to mesic niches or supported by supplemental watering.

This clustering accords with the PCA structure and fuzzy-membership rankings, reinforcing a systematic differentiation of drought strategies along hydraulic–photosynthetic–metabolic axes. For restoration design, Group I taxa are recommended as priority pioneers in drought-prone zones; Group II species fit transitional or managed-water sites; Group III should be introduced sparingly and with risk mitigation.

## 4 Discussions

### 4.1 Hydraulic safety and functional strategies

Drought tolerance in hot–dry systems aligns along a safety–efficiency continuum, with tolerant taxa prioritizing conservative water transport and sensitive taxa trading safety for short-term gains. This pattern is consistent with the classic hydraulic safety versus resource-acquisition trade-off (Tomlinson et al., 2014). In our panel, species classified as more drought tolerant buffered water transport and maintained tissue function under the most severe deficit, whereas sensitive species displayed coupled structural and physiological decline.

Morphological and physiological coordination positioned shrubs toward conservative operation while acquisitive herbs lost function under peak stress. At DS50, shrubs such as *Caryopteris forrestii* (and the shrub guild more generally) limited structural densification and preserved an operational photosynthetic apparatus, reflected by SLA retained within functional ranges and Fv/Fm remaining in the suboptimal but largely operational band (≥0.60–0.70), in contrast to sensitive herbs such as *Arthraxon lanceolatus* that showed sharp SLA contraction and photochemical impairment (Rees et al., 2010; Hunt et al., 2002; Hoffmann and Poorter, 2002). Rather than universally “low” LDMC, tolerant taxa expressed smaller drought−induced increases in LDMC—consistent with maintaining tissue pliability that supports turnover and post-drought repair (Poorter, 1992). Most species increased root:shoot ratio with drying—especially shrubs—indicating strategic belowground allocation to stabilize plant water status in arid substrates (Pons and Poorter, 2014). Taken together, these coordinated adjustments place tolerant species toward a conservative end of the spectrum that safeguards water balance and photochemistry, whereas sensitive herbs express acquisitive construction that performs well in wet windows but erodes under sustained deficit (Enquist et al., 1999).

Hydraulic thresholds confirmed wider safety margins in tolerant taxa and tighter operating domains in sensitive herbs. Tolerant species combined more negative P50 with wider hydraulic safety margins (HSM), retaining a buffer against runaway cavitation at peak stress (Wang et al., 2018). For example, *Caryopteris forrestii* reached substantially more negative P50 than *Arthraxon lanceolatus*, indicating stronger embolism resistance and lower mortality risk under severe drought (Tan et al., 2024). The sustained HSM during DS50 is consistent with the principle that drought−adapted woody plants operate with larger safety margins than herbs that approach critical tensions (Joshi et al., 2022; Huang et al., 2025).

Operating modes diverged under drought, with efficiency−first herbs favoring capacity in benign periods but incurring failure risk as soils dried (Zhang et al., 2017). Fast−growing herbs often exhibited higher instantaneous conductance and, at times, higher Kh under favorable conditions; yet this capacity co−occurred with less negative P50, rendering transport steeper and more failure−prone with progressing deficit (Carminati et al., 2020). In our experiment, sensitive herbs accumulated PLC rapidly under DS40–DS50 and showed incomplete recovery, whereas safety−first shrubs preserved transport and photochemistry and rebounded more quickly after rewatering—consistent with hydraulic design principles in which soil–plant hydraulics set stomatal behavior and failure domains (Luo et al., 2021; Lockhart et al., 2014). Mechanistically, conduit design and vulnerability tune the balance between short−term efficiency and long−term reliability along the hot–dry valley gradient.

### 4.2 Physiological resilience and recovery mechanism

Resilience during dry−down was initiated by osmotic adjustment that stabilized water status as soils dried. With increasing drought severity, leaf proline (Pro) and soluble sugars (SS) rose markedly, lowering cellular osmotic potential and sustaining water uptake under progressively drier soils (Mencuccini et al., 2019). Proline responded rapidly—often preceding other biochemical shifts—and served as an early stress sentinel in tolerant taxa; *Caryopteris forrestii* showed >80% Pro elevation at DS50 relative to the control, evidencing strong osmotic regulation capacity (Haverd et al., 2017).

Osmotic protection interacted with antioxidative defenses to contain oxidative damage, but buffering capacity was finite in sensitive species. Osmolyte build−up was positively associated with superoxide dismutase (SOD) activity, indicating coordinated enhancement of osmotic protection and reactive oxygen species detoxification (Gao et al., 2023). Under extreme stress, several sensitive species exhibited declining SOD coupled with sharp increases in malondialdehyde (MDA), consistent with lipid peroxidation and incipient irreversible damage.

Tolerant species preserved photosynthetic function and water−use economy during drought and recovered faster after rewatering. Stability reflected tight stomatal control and improved instantaneous water−use efficiency (WUE), which preserved carbon assimilation despite constrained conductance (Torres-Ruiz et al., 2024; Jain et al., 2024). For example, *Sophora davidii* sustained Pn ≥ 4.3 μmol m^-2^ s^-1^ at peak stress, whereas sensitive species approached photosynthetic standstill.

Photoprotective energy dissipation buffered PSII integrity at high stress. To avoid photoinhibition, tolerant species upregulated non−photochemical quenching (qN); *Elsholtzia capituligera* showed pronounced qN increases under severe drought (Ma et al., 2023). Concomitantly, these species retained relatively high Fv/Fm (≈0.60–0.70) versus ≈0.40 in sensitive taxa, indicating that PSII reaction centers remained largely functional under stress.

Rapid antioxidant enzyme induction limited molecular damage and supported functional reversibility (Takahashi and Badger, 2011). In tolerant species, induction of SOD and peroxidase (POD) curtailed MDA accumulation and protected photosynthetic proteins and membranes; following rehydration, these taxa exhibited more complete rebounds of Pn and chlorophyll−fluorescence parameters, whereas sensitive species frequently showed excessive stomatal closure, insufficient energy dissipation, weak antioxidant mobilization, and poor recovery (Santini et al., 2024).

Coordinated use efficiencies linked carbon, nutrients, and water to underpin drought performance. Because drought often co−occurs with nutrient limitation, high nitrogen− and phosphorus−use efficiencies (NUE, PUE) are advantageous for metabolic maintenance (de Sousa Leite et al., 2023). In our panel, *Caryopteris forrestii* and *Excoecaria acerifolia* achieved elevated PNUE (up to 42%) and PUE (>35%) that remained comparatively stable under drought; across species, NUE, PUE, WUE, and Pn were positively correlated, indicating coordinated regulation of carbon, nutrient, and water pathways (Gleason et al., 2017).

Phenology can confound late−experiment trajectories, and time−matched controls clarify recovery versus growth. All species began at comparable age in the vegetative stage; inferences about “recovery” compare droughted plants from DS50→RE10 with the natural change in controls over the same window (DS50→DS60). Where droughted plants do not exceed the control’s concurrent increase, we attribute late−stage rises primarily to growth rather than recovery (Skelton et al., 2017). Apparent upticks that lack concordant stress−response signals (e.g., no co−elevation in Pro/SOD or qN) are treated conservatively as phenology−driven variation (Li et al., 2021).

Physiological resilience emerges from integrated management of water status (osmolytes, stomatal control), energy balance (qN−mediated dissipation), and redox homeostasis (antioxidant enzymes), reinforced by efficient nutrient use. Benchmarking against time−matched controls prevents conflation of recovery responses with life−cycle effects and strengthens causal attribution to drought versus growth.

### 4.3 Hydraulic physiological co adaptation

Multivariate structure showed that drought tolerance is an emergent property of coordinated hydraulic and physiological shifts rather than single−trait responses. Principal component analysis (PCA) and pairwise correlations identified a dominant axis (PC1) loading on leaf water status (RWC, TLP), osmotic adjustment (Pro, SS), photosynthetic performance (Pn, Gs, ETR), and antioxidant capacity (SOD), explaining the largest share of variance (>30%), consistent with trait integration under stress (Xu et al., 2021).

Hydraulic restoration governed the pace of photosynthetic rebound after rewatering. As soil water declined, plants lowered leaf water potential and accumulated osmolytes to sustain turgor—hydraulic adjustments that buffered photosynthetic impairment; upon rewatering, rapid increases in RWC preceded stomatal reopening and Pn recovery, underscoring that photosynthetic resilience depends on hydraulic repair rather than physiological regulation alone (Li et al., 2023b).

Trait−network topology identified central hubs and failure domains linking transport, photochemistry, and redox balance. Pn and Gs were positively associated with Kh and with more negative P50, but negatively associated with PLC, consistent with embolism−induced transport limitation. SOD tracked RWC and was inversely related to MDA; its association with Pro/SS was stress−dependent—co−elevated at moderate drought but decoupling or turning negative at peak stress when ROS burden exceeded enzymatic capacity (**Figure 8**). Within the network, Kh, P50, and TLP formed central hubs that connected hydraulic status to photochemistry and redox balance, whereas PLC and qN delineated a negatively associated core indicative of failure domains where hydraulic disconnection and photoprotective dissipation coincide (Wang et al., 2025).

Integrated scoring resolved three functional types with distinct risk profiles useful for restoration planning. Weighted PCA scores and fuzzy−membership indices yielded: (i) Hydraulic−Safe & Physiologically−Resilient—*Caryopteris forrestii*, *Elsholtzia capituligera*, *Sophora davidii*, *Rumex hastatus*: more negative P50, wider HSM, higher Fv/Fm under stress, stronger WUE, and rapid post−rewatering recovery; (ii) Photosynthetically Fast−Growing—*Vitex negundo*, *Excoecaria acerifolia*: high Pn/ETR during favorable windows but less negative P50 and narrower HSM, implying higher vulnerability to prolonged or extreme drought; (iii) Hydraulically & Physiologically Vulnerable—*Ceratostigma minus*, *Incarvillea arguta*, *Artemisia vestita*, *Arthraxon lanceolatus*: consistently lower Kh, WUE, and SOD with higher PLC/qN under stress and incomplete recovery.

Applied implications. From a restoration perspective, species with high composite scores—particularly *Rumex hastatus*, *Caryopteris forrestii*, and *Sophora davidii*—should be prioritized as core planting materials because they combine hydraulic safety with robust physiological resilience. Lower-ranked herbs can still be deployed tactically (e.g., in mesic microsites or as early-successional cover) but warrant caution under intensifying drought regimes. By operationalizing hydraulic and physiological coordination within a unified framework, our approach improves species matching to site water balance and enhances the stability of revegetation outcomes in drought-prone drylands.

## 5 Conclusion

Using a controlled drought gradient followed by rewatering, we show how ten representative dry–hot valley species coordinate hydraulics, physiology, photosynthesis, and nutrient use. Three concise insights emerge.

1. Life‐form divergence is clear: shrubs generally combine more negative P50 and wider hydraulic safety margins with faster post-drought rebound (a conservative strategy), whereas herbs rely on acquisitive leaf economics (higher SLA and WUE) but operate with narrower safety margins under extreme drought.
2. resilience reflects hydraulic–physiological coordination: hydraulic thresholds (P50, TLP, Kh) covary with osmotic adjustment, antioxidant defenses, and photochemical stability, enabling tolerant species to limit damage and recover efficiently after rewatering.
3. An integrated classification (PCA, clustering, fuzzy scoring) resolves functional groups useful for restoration; *Rumex hastatus*, *Caryopteris forrestii*, and *Sophora davidii* emerge as priority pioneers. For rapid, field-ready screening, we recommend a minimal diagnostic panel—P50, TLP, HSM, and post-rewatering Fv/Fm recovery—optionally complemented by acquisitive leaf traits (e.g., SLA, PNUE) to place species along the safety–efficiency–resilience spectrum and support evidence-based revegetation in increasingly variable hydroclimates.

## Data Availability

The original contributions presented in the study are included in the article/**Supplementary Material**. Further inquiries can be directed to the corresponding author.
